# SOX2 and PI3K Cooperate to Induce and Stabilize a Squamous-Committed Stem Cell Injury State during Lung Squamous Cell Carcinoma Pathogenesis

**DOI:** 10.1371/journal.pbio.1002581

**Published:** 2016-11-23

**Authors:** Bo Ram Kim, Emily Van de Laar, Michael Cabanero, Shintaro Tarumi, Stefan Hasenoeder, Dennis Wang, Carl Virtanen, Takaya Suzuki, Bizhan Bandarchi, Shingo Sakashita, Nhu An Pham, Sharon Lee, Shaf Keshavjee, Thomas K. Waddell, Ming-Sound Tsao, Nadeem Moghal

**Affiliations:** 1 Princess Margaret Cancer Centre, University Health Network, Toronto, Ontario, Canada; 2 Department of Medical Biophysics, University of Toronto, Toronto, Ontario, Canada; 3 Division of Thoracic Surgery, University Health Network, University of Toronto, Toronto, Ontario, Canada; B.C. Cancer Agency, CANADA

## Abstract

Although cancers are considered stem cell diseases, mechanisms involving stem cell alterations are poorly understood. Squamous cell carcinoma (SQCC) is the second most common lung cancer, and its pathogenesis appears to hinge on changes in the stem cell behavior of basal cells in the bronchial airways. Basal cells are normally quiescent and differentiate into mucociliary epithelia. Smoking triggers a hyperproliferative response resulting in progressive premalignant epithelial changes ranging from squamous metaplasia to dysplasia. These changes can regress naturally, even with chronic smoking. However, for unknown reasons, dysplasias have higher progression rates than earlier stages. We used primary human tracheobronchial basal cells to investigate how copy number gains in *SOX2* and *PIK3CA* at 3q26-28, which co-occur in dysplasia and are observed in 94% of SQCCs, may promote progression. We find that SOX2 cooperates with PI3K signaling, which is activated by smoking, to initiate the squamous injury response in basal cells. This response involves *SOX9* repression, and, accordingly, SOX2 and PI3K signaling levels are high during dysplasia, while SOX9 is not expressed. By contrast, during regeneration of mucociliary epithelia, PI3K signaling is low and basal cells transiently enter a SOX2^Lo^SOX9^Hi^ state, with SOX9 promoting proliferation and preventing squamous differentiation. Transient reduction in SOX2 is necessary for ciliogenesis, although SOX2 expression later rises and drives mucinous differentiation, as SOX9 levels decline. Frequent coamplification of *SOX2* and *PIK3CA* in dysplasia may, thus, promote progression by locking basal cells in a SOX2^Hi^SOX9^Lo^ state with active PI3K signaling, which sustains the squamous injury response while precluding normal mucociliary differentiation. Surprisingly, we find that, although later in invasive carcinoma SOX9 is generally expressed at low levels, its expression is higher in a subset of SQCCs with less squamous identity and worse clinical outcome. We propose that early pathogenesis of most SQCCs involves stabilization of the squamous injury state in stem cells through copy number gains at 3q, with the pro-proliferative activity of SOX9 possibly being exploited in a subset of SQCCs in later stages.

## Abstract 2

A study of tracheobronchial basal cells reveals a mechanism by which lung squamous cell carcinoma is almost universally initiated by exploitation of a stem cell injury response involving SOX2 and PI3K.

## Author Summary

Squamous cell carcinoma (SQCC) is a deadly and common form of lung cancer. How it develops from stem cells is poorly understood. SQCCs predominantly arise in bronchial epithelia, likely from basal cells, stem cells that normally generate mucinous and ciliated cells. Smoking, however, causes normally quiescent basal cells to proliferate and generate protective squamous epithelia. Continuous smoking eventually causes precancerous changes and, ultimately, SQCC. However, some precancerous changes regress to normal epithelia, suggesting that the natural stem cell injury response is not sustainable indefinitely. Here, we describe how the SOX2 transcription factor and PI3K signaling, which is activated by smoking, induce the squamous injury response in basal cells. We also provide evidence that regeneration of mucociliary epithelia after injury requires basal cells to enter a period of low SOX2 expression and PI3K signaling. Ninety-four percent of SQCCs have copy number gains in chromosome 3 that affect *SOX2* and *PIK3CA*, a catalytic PI3K subunit. These gains occur at a point when precancerous tissue has an increased likelihood of progressing to SQCC. Our data suggest that SQCC is genetically initiated by events that sustain a normally self-limiting injury state, which forces stem cells to proliferate indefinitely in the presence of smoking-associated mutagens.

## Introduction

It has been suggested that most cancers arise from normal stem cells [1,2]. Mounting evidence supports this being the case for certain leukemias, in which stem cell/progenitor-specific mechanisms are emerging that involve blocks in differentiation and enhanced self-renewal [3,4]. However, for many carcinomas, it has been challenging to elucidate similar mechanisms, as the normal stem cell biology of many tissues is not well characterized. Thus, to a large extent, the mechanisms through which human stem cells are transformed are poorly understood.

Lung cancer is the worldwide leading cause of cancer mortality [5], and, in general, pathogenesis of all types is poorly understood. Squamous cell carcinoma (SQCC) is the second most common form [6], and its pathogenesis appears to depend on a critical change in the behavior of the stem cells from which the disease likely initiates. Most SQCCs arise in the bronchial epithelium [7], which is part of a contiguous epithelium that extends from the nasopharynx through the trachea and bronchi. The major cell types include basal, ciliated, and secretory/mucinous cells, with basal cells being the stem cells for these lineages [8–12]. Basal cells are also thought to be the main origin for SQCCs because virtually all SQCCs express basal cell markers [13–15]. Under homeostatic conditions, most cells in the epithelium are quiescent, with only 1% cycling [16]. However, vitamin A deficiency, denudation, or smoking—the greatest SQCC risk factor [17,18]—induces a stereotypical metaplastic response in basal cells, which causes replacement of the mucociliary epithelium with a hyperproliferative squamous epithelium [19–26]. This response is reversible, as vitamin A resupplementation or injury/smoking cessation restores mucociliary differentiation [20,24,27]. Many squamous metaplasias also regress in chronic active smokers [28–30], suggesting that the squamous response cannot naturally be maintained indefinitely.

Longitudinal studies have shown, however, that some squamous metaplasias progress to SQCC in a stepwise manner through low and high grade dysplasia and through carcinoma in situ [28,31]. Although low grade dysplasias regress at a high rate, high grade dysplasia and carcinoma in situ mark a different phase of progression, as these later stages have more genetic alterations, lower rates of regression, and higher rates of progression to SQCC [28,32–36]. Thus, SQCCs appear to arise through phenotypic progression of the squamous injury response, with genetic events possibly stabilizing a dysregulated injury state and preventing regression. One of the earliest genetic events is *SOX2* amplification, which is common in high grade dysplasias and is associated with greater progression to SQCC [37–40]. Ultimately, *SOX2* copy number gains are found in 94% of SQCCs (54% amplification/40% lower copy number gain only, provisional TCGA (The Cancer Genome Atlas) data, www.cbioportal.org) [41,42]. Although several studies support *SOX2* being a driver [41,43,44], it resides in a broad amplicon spanning 3q26-28, which includes other oncogenes such as *PIK3CA* [41,42]. How *SOX2* amplification may specifically promote progression of premalignant squamous lesions at the expense of mucociliary differentiation is a mystery, especially considering its wide-ranging roles in a variety of stem cells [45–49].

Although there have been several attempts to genetically model SQCC pathogenesis in mice [44,50,51], it is unclear to what extent these models faithfully recapitulate human disease pathogenesis, and a stem cell-based mechanism is still lacking. In all cases, which included functionally distinct drivers such as *Sox2* overexpression, *Pten* loss, and *Kras* mutation, *Lkb1* inactivation was necessary for SQCC generation, and in one model, SQCC was generated in distal airways through transdifferentiation of adenocarcinoma (ADC) [50]. However, in human lung cancer, *LKB1* DNA alterations are infrequent in SQCCs and more common in ADCs (3% of SQCCs and 19% of ADCs, provisional TCGA data, www.cbioportal.org) [42,52], and SQCCs generally do not arise in distal airways. These findings question whether differences between human and mouse airway epithelia affect mechanisms of SQCC pathogenesis. Indeed, although in the murine tracheal epithelium, basal cells are stem cells [11,12], 50% of their progeny are club cells (previously known as Clara cells) [53]. Club cells are secretory cells that are the major stem cell population in the bronchiolar epithelium, but they can also contribute to renewal in the tracheal epithelium, especially after injury [12,54,55]. However, in human bronchial epithelia, the main site of SQCC carcinogenesis [7], club cells are not found (although they are found in human bronchiolar epithelia) [56]; instead, there are many more mucinous cells [53,57].

We thus modeled SQCC pathogenesis in the putative stem cell origin of the disease, using primary human tracheobronchial basal cell cultures. These cultured human basal cells regenerate normal ciliated and mucinous lineages when grown in denuded rat tracheal xenografts or on porous filters in air-liquid-interface (ALI) cultures, where they are fed basolaterally and apically exposed to air [8,58–62]. In ALI cultures, differentiated epithelia recapitulate in vivo ultrastructure and functional properties, including mucous production, ion transport, bacterial defense, and movement of airway surface liquid by cilia. Using these cultures, we describe a mechanism by which *SOX2* amplification and PI3K signaling cooperate to stabilize a hyperproliferative stem cell injury state that promotes squamous metaplasia at the expense of normal mucociliary differentiation. This mechanism potentially explains selection of *SOX2* and *PIK3CA* coamplification during SQCC pathogenesis and how it may promote progression of high-grade dysplasias by altering the behavior of stem cells.

## Results

### In Normal Quiescent Mucociliary Epithelia, Tracheobronchial Basal Cells Express Similar Levels of SOX2 as SQCCs

**Fig 1:**
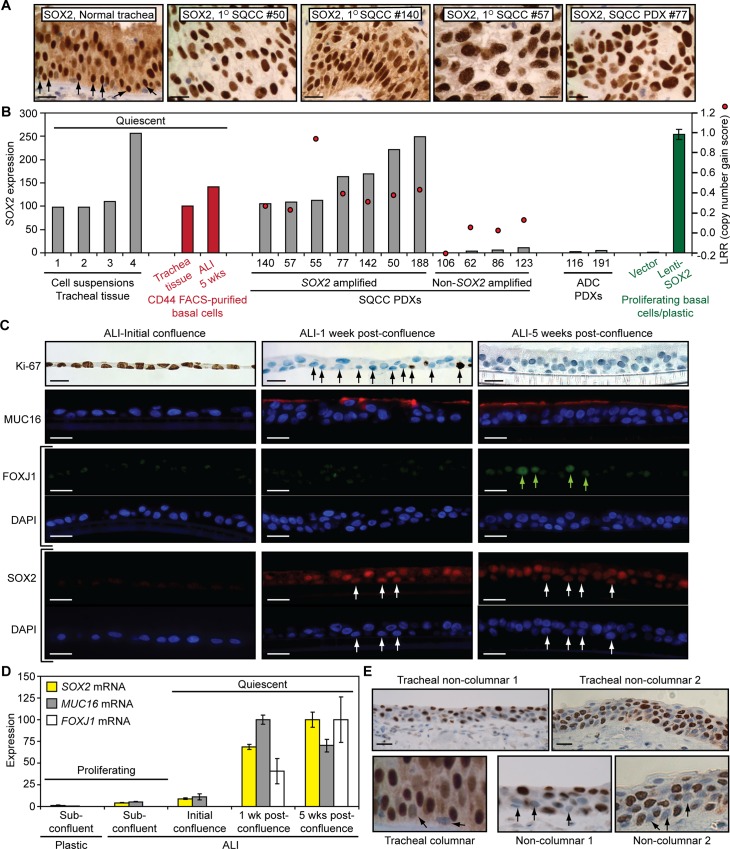
During mucociliary differentiation of tracheobronchial basal cells, SOX2 expression varies from low to high. (A) SOX2 immunohistochemistry (IHC) in normal native human tracheobronchial epithelia and *SOX2*-amplified primary patient lung SQCCs and SQCC patient-derived xenografts (PDXs). Arrows point to some basal cells. (B) qRT-PCR quantification of *SOX2* expression in normal tracheobronchial epithelial cells and SQCCs. Tracheobronchial cell suspensions and FACS-purified basal cells from these suspensions were derived from tissue without culturing. ADC = primary patient lung adenocarcinoma. All data, with the exception of “Proliferating basal cells on plastic” (green), are from individual biological replicates, which were generated from duplicate qRT-PCR technical replicates. Tracheobronchial basal cells proliferating on plastic were infected with Lenti-SOX2 or empty vector, with mean expression ± standard error of the mean (SEM) from three biological replicate experiments shown. Control empty vector did not alter *SOX2* expression relative to untransduced basal cells (not shown). LRR = log likelihood ratio quantification of *SOX2* gene copy number. Expression is normalized to normal tracheal suspension #1, which was assigned a value of 100. (C, D) SOX2 and mucociliary lineage marker expression during tracheobronchial basal cell differentiation in air-liquid-interface (ALI) cultures. (C) Immunofluorescence staining of SOX2 and lineage marker expression. White arrows point to some basal cells and green arrows mark FOXJ1+ cells. (D) qRT-PCR analysis of *SOX2* and lineage marker expression. Data are plotted relative to the time point with the maximal expression of the gene, which was given a value of 100. Means ± SEM from three replicates are shown. (E) SOX2 IHC in metaplastic areas of native human tracheobronchial epithelia. Scale bars are 20 μm. All plotted numerical data are in S1 Data.

In SQCCs, *SOX2* copy number gains are correlated with increased *SOX2* expression (S1A Fig) [41,42,63]. However, the extent to which SOX2 is overexpressed relative to normal basal cells has not been reported. Surprisingly, we found that *SOX2*-amplified SQCC primary patient tumors and patient-derived xenografts (PDXs) expressed similar levels of SOX2 protein as normal cells in native human tracheobronchial epithelia, including basal cells (Figs 1A and S1B). In support of this finding, quantitative reverse transcription-polymerase chain reaction (qRT-PCR) analysis indicated that freshly harvested cell suspensions from tracheobronchial tissue, as well as FACS-purified basal cells from these suspensions, expressed equivalent levels of *SOX2* mRNA as *SOX2*-amplified SQCC PDXs (Fig 1B). These normal levels of expression were also much higher than in non-*SOX2*-amplified SQCC and ADC PDXs (Fig 1B).

### During Regeneration of Mucociliary Epithelia, SOX2 Levels Transiently Decline

The similar levels of *SOX2* expression between basal cells in normal mucociliary epithelia and *SOX2*-amplified SQCCs raise several considerations. First, at some point during SQCC progression, *SOX2* promoter activity may decline, necessitating amplification to sustain high levels of expression. Second, because amplification would be expected to raise expression of a driver to levels higher than in the normal cell of origin, if SOX2 is, indeed, a driver, there should be a period during SQCC pathogenesis when SOX2 is expressed at lower levels in normal basal cells. However, this period may not have been captured in our snapshot analysis of healthy tracheobronchial epithelium. Notably, SQCCs do not spontaneously arise in healthy epithelia, but do arise after years of smoking, and *SOX2* amplification is commonly detected in dysplasia, in which it is associated with a higher rate of progression to SQCC [37–40]. These data suggest that a decline in SOX2 expression may be part of the mechanism of regenerating mucociliary epithelia following injury, and that *SOX2* amplification may be selected to counteract this decline and, hence, prevent regression.

To determine if SOX2 expression varies during mucociliary differentiation, we used ALI cultures. Although in denuded tracheas, basal cells first undergo squamous differentiation before regenerating mucociliary epithelia [20], ALI culture conditions, which include retinoic acid, promote mucociliary differentiation without a prior phase of squamous metaplasia [58]. Thus, these cultures can be used to study basal cells as they synchronously undergo mucociliary differentiation. To establish such cultures, we first isolated basal cells from normal tracheobronchial tissue and expanded them on plastic dishes in an undifferentiated state. We confirmed that these plastic cultures consisted only of basal cells by quantifying basal cell lineage marker expression (TP63 [64], KRT5 [65], and CD44 [66]) (S2A and S2B Fig). They also retained stem cell activity, as evidenced by morphological and molecular differentiation into mucinous (MUC16+ [67]) and ciliated (FOXJ1+ [68]) cells when placed in ALI culture and denuded rat tracheal xenografts (Figs 1C and S2C). During expansion on plastic, *SOX2* expression dramatically declined relative to the quiescent state in normal tissue (Fig 1B, “vector” plastic cultures). This low level of *SOX2* expression persisted during the early proliferative phase of ALI culture (Fig 1D). However, during mucociliary differentiation after the onset of quiescence, SOX2 expression in ALI culture returned to normal tissue levels (Fig 1B–1D). Thus, in ALI culture, a SOX2^Lo^ state precedes mucociliary differentiation. Based on these data, we re-examined SOX2 expression in native human tracheobronchial tissue to see if SOX2^Lo^ cells might also be present in vivo. By immunohistochemistry (IHC), rare SOX2^Lo^ basal cells were present in mucociliary epithelia, and appeared to be enriched in non-columnar areas, which could be undergoing mucociliary differentiation (Fig 1E). We confirmed that these SOX2^Lo^ cells were, indeed, basal cells by costaining for the basal cell marker KRT5 (S1C Fig). Thus, *SOX2* amplification could prevent regression of dysplasias by preventing generation of a SOX2^Lo^ state that might be necessary for normal mucociliary differentiation.

### Precocious SOX2 Expression in Proliferating Basal Cells Induces Hyperproliferative Squamous Metaplasia and Inhibits Ciliogenesis

**Fig 2:**
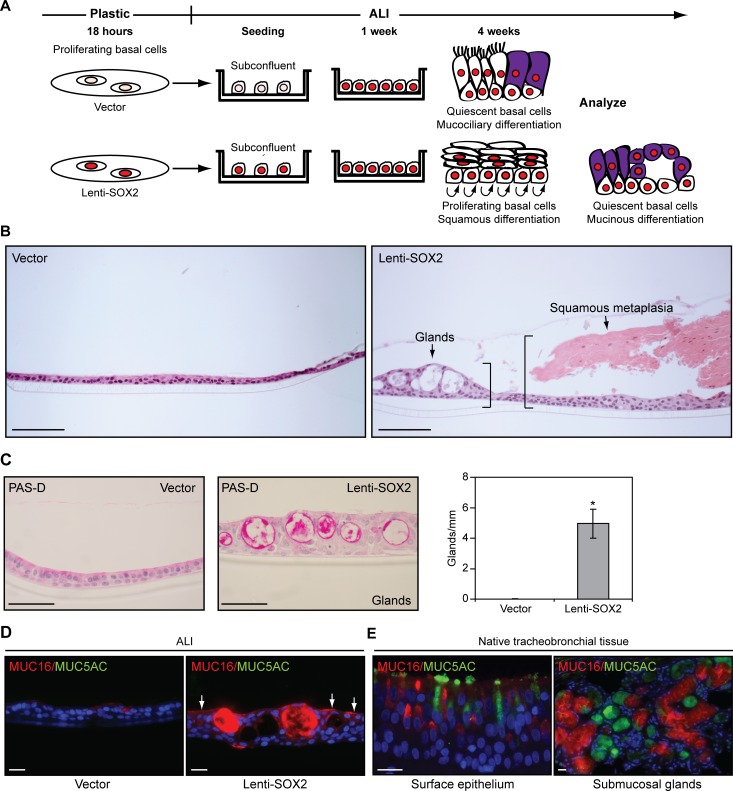
Precocious SOX2 expression in proliferating tracheobronchial basal cells enhances mucinous differentiation. (A) Experimental design. SOX2^Lo^ tracheobronchial basal cells proliferating on plastic were infected overnight with Lenti-SOX2 or empty vector, seeded subconfluently at ALI, and examined after 5 wk. (B) Hematoxylin and eosin (H&E) staining showing ectopic induction of glandular-like areas and squamous metaplasia by Lenti-SOX2. (C) PAS-D (periodic acid Schiff-diastase) staining for mucins. Glandular differentiation was quantified by scoring 6–7 cm of epithelia from multiple sections derived from three replicates. Mean ± SEM is shown. Plotted numerical data are in S1 Data. (D) MUC16 and MUC5AC mucin staining. Arrows point to non-glandular cells with increased MUC16 expression relative to vector control cultures. Due to high MUC16 expression in Lenti-SOX2 cultures, the exposure time was shorter than in Fig 1C. (E) MUC16 and MUC5AC mucin staining in native human tracheobronchial tissue. Scale bars are 100 μm (B), 50 μm (C), and 20 μm (D, E). Significance was calculated using a two-tailed *t* test. *p = 0.006.

To investigate whether precocious SOX2 expression, which would be caused by *SOX2* amplification, affects mucociliary differentiation, we expressed SOX2 in proliferating basal cell ALI cultures before its normal time of expression. *SOX2* was expressed from a lentiviral vector at the same mRNA levels seen in normal native tissue and *SOX2*-amplified SQCCs (Fig 1B, Lenti-SOX2). Proliferating basal cells were infected on plastic dishes overnight, seeded subconfluently into ALI cultures, and differentiated over 5 wk (Fig 2A). Histological analyses of the 5-wk cultures indicated that precocious SOX2 expression enhanced mucinous differentiation and induced squamous metaplasia (Fig 2B). Lenti-SOX2 induced ectopic gland-like structures that were confirmed mucinous by periodic acid-Schiff (PAS) reactivity (Fig 2C). It also increased MUC16 expression relative to control cultures (Fig 2D), wherein endogenous SOX2 expression was naturally correlated with MUC16 expression (Fig 1C and 1D). Although MUC16+ cells have been noted in the tracheal epithelium [67], we now show that these cells do not express the canonical goblet cell marker MUC5AC (Fig 2D and 2E) and, hence, are a distinct subtype of mucinous lineage that is induced by SOX2.

**Fig 3:**
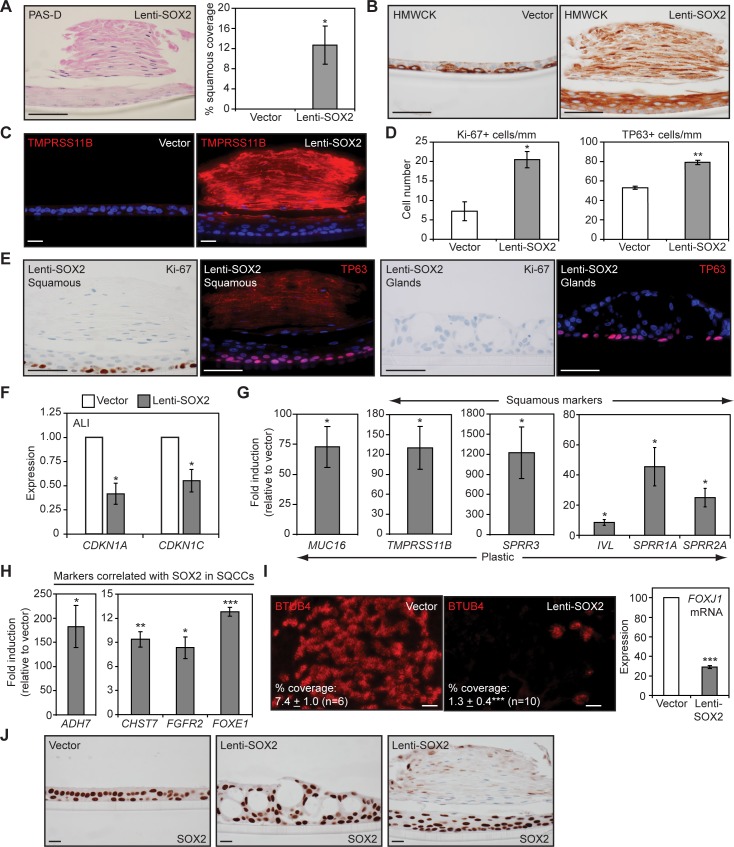
Precocious SOX2 expression in proliferating tracheobronchial basal cells induces hyperplastic squamous metaplasia and inhibits ciliogenesis. (A–F, I, J) For all ALI data, SOX2^Lo^ proliferating tracheobronchial basal cells were infected with Lenti-SOX2 or control vector and grown as described in Fig 2A. All ALI epithelia were analyzed after 5 wk of ALI culture. (A) PAS-D staining showing absence of mucin expression in areas of squamous metaplasia. Squamous metaplasia was quantified by scoring 6–7 cm of epithelia from multiple sections derived from three replicates. Mean ± SEM is shown. Significance was calculated by a two-tailed *t* test. *p = 0.03. (B) HMWCK (high molecular weight cytokeratin) staining, whose expression in upper layers marks squamous stratifying epithelia. (C) Staining for TMPRSS11B, a marker of squamous epithelia. (D, E) Lenti-SOX2 induces hyperplasia. ALI sections were stained for Ki-67 and TP63. At least eight sections were scored per replicate from three replicates. Means ± SEM are shown. Significance was calculated by two-tailed *t* tests. *p = 0.01, **p = 0.0007. (F) qRT-PCR analysis of *CDKN* expression in ALI cultures. Means ± SEM from four replicates are shown. Data are normalized to expression in vector-transduced cultures, which was assigned a value of 1. Significance was calculated by paired two-tailed *t* tests. *p = 0.012 (*CDKN1A*), 0.03 (*CDKN1C*). (G, H) Enforced SOX2 expression is sufficient to induce mucinous, squamous, and SQCC-like differentiation in tracheobronchial basal cells growing on plastic. Basal cells were infected with Lenti-SOX2 or control empty vector and marker expression was measured by qRT-PCR after 5 d. Data are normalized to expression in vector-transduced cultures, which was assigned a value of 1. Means ± SEM from three to five replicates are shown. Significance was calculated by paired two-tailed *t* tests. (G) *p = 0.02 (*MUC16*), 0.02 (*TMPRSS11B*), 0.05 (*SPRR3*), 0.02 (*IVL*), 0.04 (*SPRR1A*), 0.02 (*SPRR2A*). (H) *p = 0.01 (*ADH7*), 0.01 (*FGFR2*), **p = 0.003, ***p = 0.00003. (I) Lenti-SOX2 inhibits ciliogenesis. ALI cultures were stained *enface* for BTUB4 (a component of cilia), and expression of the ciliogenic *FOXJ1* transcription factor was quantified by qRT-PCR, with means ± SEM from four replicates shown. For BTUB4 expression, significance was calculated by a two-tailed *t* test. ***p = 0.000007. For *FOXJ1* expression, data are normalized to levels in vector-transduced cultures, which was assigned a value of 100, and significance was calculated by a paired two-tailed *t* test. ***p = 0.00007. (J) SOX2 IHC in lentivirally-infected ALI cultures. Scale bars are 50 μm (A, B, E) and 20 μm (C, I, J). All plotted numerical data are in S1 Data.

Although squamous differentiation is normally suppressed in ALI cultures [58], Lenti-SOX2 induced squamous metaplasia in some epithelial regions, as evidenced by histological stratification and expression of high molecular weight keratins (Fig 3A and 3B), which are normally abundant in upper layers of squamous, but not columnar, epithelia [69]. Squamous metaplasias also expressed TMPRSS11B (Fig 3C), a marker of squamous epithelia [70], and contained hyperplastic basal cells (Fig 3D and 3E). The latter phenotype recapitulates the hyperproliferation of squamous metaplasias in smokers [21] and was correlated with reduced expression of cell cycle inhibitors that include a SOX2 target in SQCCs, *CDKN1A* (Fig 3F) [71]. When maintained on plastic, which does not normally support mucinous or squamous differentiation, Lenti-SOX2 was sufficient to induce expression of *MUC16* and well-established markers of the cross-linked envelopes of squamous epithelia, including involucrin (IVL) and small proline-rich proteins (SPRRs) (Fig 3G). In plastic cultures, Lenti-SOX2 also induced expression of genes correlated with *SOX2* levels in patient SQCCs (Fig 3H) [41], providing further evidence that the squamous response on plastic recapitulates features of SQCC pathogenesis.

Precocious SOX2 expression also affected ciliogenesis. In ALI culture, Lenti-SOX2 reduced the number of ciliated cells, as evidenced by fewer BTUB4-expressing cells, and decreased *FOXJ1* expression (Fig 3I). Given that SOX2 expression is ultimately seen in ciliated cells (e.g., S1B Fig), there appears to be an early period when SOX2 levels must be below a certain threshold for ciliated cell fate commitment to occur. Notably, by the time control epithelia had completed mucociliary differentiation, they expressed similar levels of SOX2 as Lenti-SOX2-transduced epithelia (Fig 3J). This finding supports squamous metaplasia in ALI culture being driven by an alteration in the timing of SOX2 expression rather than by supraphysiologic levels, and suggests that distinct cellular contexts (e.g., associated with proliferation versus quiescence) may alter the stem cell response to similar levels of SOX2.

### PI3K Signaling Is Necessary for the Squamous Response to SOX2

**Fig 4:**
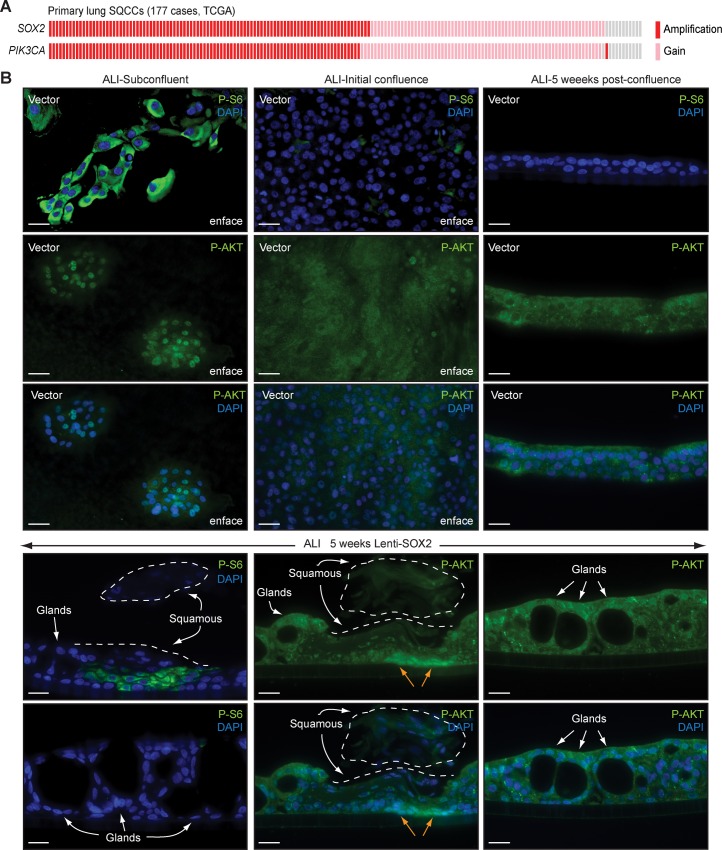
PI3K signaling is high in proliferating basal cells and in squamous differentiating epithelia. (A) *PIK3CA* is co-amplified with *SOX2* at 3q26-28 in lung SQCCs. *SOX2* and *PIK3CA* copy number variation data for 177 primary patient SQCCs from the TCGA. Numerical data are in S1 Data. (B) PI3K activity in ALI cultures of tracheobronchial basal cells. Basal cells were infected with Lenti-SOX2 or control vector and grown at ALI, as described in Fig 2A. Cultures were stained for phospho-Ser240/244-S6 (P-S6) or phospho-Thr308-AKT (P-AKT) at the indicated times. Dotted lines outline areas of squamous metaplasia, including upper differentiated layers that had detached during sectioning. Orange arrows mark basal cells in squamous metaplasia that are stained positive for nuclear P-AKT. Scale bars are 20 μm.

Since squamous metaplasia is not observed in ALI cultures when SOX2 levels naturally rise after quiescence but occurs if SOX2 is precociously expressed during the proliferative phase of culture, a signal associated with this phase may cooperate with SOX2 to induce the squamous response. Cigarette smoke extract and nicotine induce activation of AKT, a downstream effector of PI3K signaling, in tracheobronchial basal cell cultures [72,73], and PI3K signaling is elevated in dysplasias when *SOX2* amplification is common [37,38,74–77]. Furthermore, AKT can phosphorylate SOX2 [78,79], and *PIK3CA*, which encodes the p110α catalytic subunit of PI3K, is in the 3q amplicon, with gains co-occurring in 99% of SQCCs with *SOX2* gains (Fig 4A) [42]. To investigate the role of PI3K signaling in squamous differentiation, we first examined its activity in proliferating versus quiescing basal cell cultures. To assess PI3K signaling, we initially focused on phosphorylation of the S6 ribosomal subunit on sites regulated by a PI3K-mTOR-S6 kinase axis [80–82]. In control ALI cultures, P-S6 was high during the proliferative SOX2^Lo^ phase but declined at initial confluence, before SOX2 levels normally rise and mucociliary differentiation occurs (Figs 4B, 1C and 1D for SOX2 expression). Nuclear accumulation of phospho-Thr308-AKT, which is PI3K-dependent [83], tracked with P-S6 and provided additional evidence that PI3K signaling is more active in proliferating rather than quiescing basal cells (Fig 4B). Consistent with the ALI data, P-S6 and nuclear P-AKT staining were also low in basal cells of quiescent native tracheal epithelia (S3 Fig). Thus, PI3K signaling is specifically active during the period when SOX2 levels are normally low and precocious SOX2 expression causes later manifestation of squamous metaplasia. Notably, by contrast to control differentiated cultures, in 5-wk Lenti-SOX2-transduced ALI cultures, PI3K signaling was active, but only in squamous metaplasias and not adjacent glandular areas (Fig 4B). This finding is consistent with premalignant squamous lesions having elevated PI3K signaling [74–77]. It also suggests that SOX2 may be able to amplify PI3K signaling in certain cell contexts such as squamous-committed cells, which has been observed in esophageal SQCC cell lines [84].

**Fig 5:**
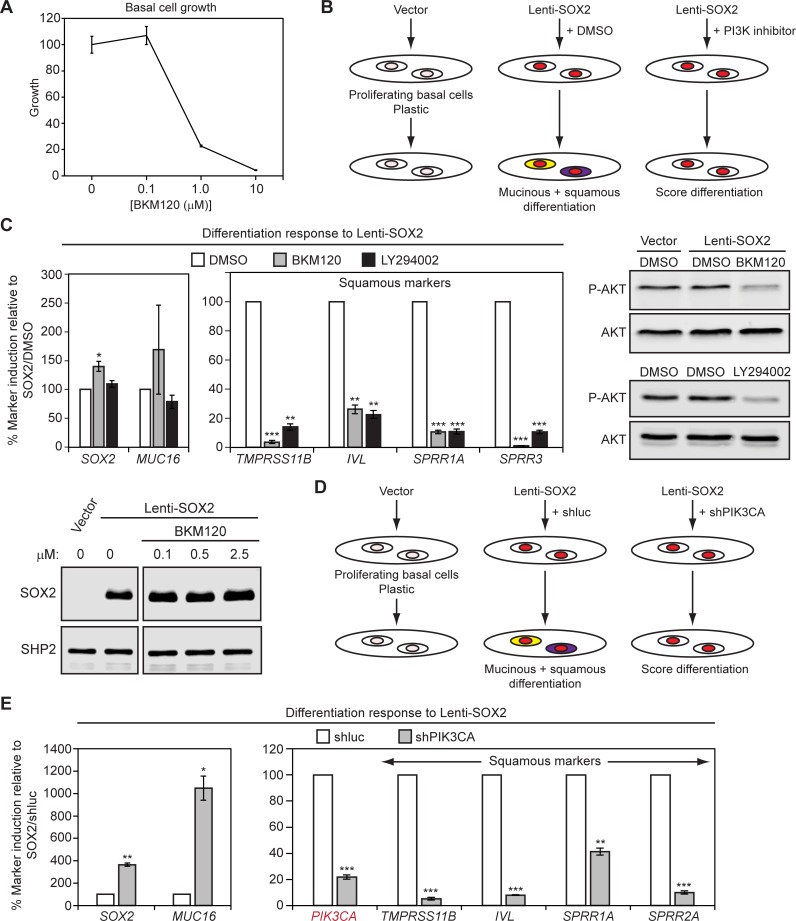
PI3K signaling is necessary for the squamous response to SOX2. (A) The PI3K inhibitor BKM120 inhibits growth of tracheobronchial basal cells. Basal cells were grown on plastic over 7 d with fresh media and drug added every other day. Growth was quantified by alamarBlue and is normalized to control cells treated with the DMSO vehicle, which was given a value of 100%. The means ± SEM from three replicates are shown. (B-E) Inhibition of PI3K signaling prevents SOX2-driven squamous differentiation. Tracheobronchial basal cells growing on plastic were infected with Lenti-SOX2 or empty vector, co-treated with the indicated drugs or shRNA viruses for 5 d, and then assayed for lineage marker expression by qRT-PCR. Fold inductions were first calculated by comparing marker expression between Lenti-SOX2 and control vector (non-SOX2)-transduced cells. Because the magnitudes of inductions sometimes varied between biological replicate experiments, fold-inductions were directly compared between matched pairs of Lenti-SOX2 (with DMSO or shluc) and PI3K-inhibited (chemical inhibitor or shPIK3CA) Lenti-SOX2-transduced cultures. The amount of marker induction in inhibitor-treated cultures was then plotted as a percentage of the induction response seen without inhibitor treatment, which was given a value of 100. (B) Schematic for the PI3K chemical inhibitor experiments. White cells represent undifferentiated basal cells. Yellow and purple depict squamous and mucinous-differentiating cells, respectively. Red denotes cells that have been transduced with Lenti-SOX2. (C) Summary of PI3K chemical inhibitor data. Lentivirally infected cultures were co-treated with 2.5 μM BKM120, 4 μM LY294002, or DMSO vehicle. Means ± SEM of three replicates are shown. Significance was calculated using paired two-tailed *t* tests. BKM120-treated *p*-values include *p = 0.04, **p = 0.004 (*IVL*), ***p = 0.0001 (*TMPRSS11B*), 0.0002 (*SPRR1A*), and 0.000002 (*SPRR3*). LY294002-treated *p*-values include **p = 0.0006 (*TMPRSS11B*), 0.001 (*IVL*), ***p = 0.0004 (*SPRR1A*), and 0.0002 (*SPRR3*). For AKT immunoblotting, lysates were prepared at 2 hr (LY294002) and 24 hr (BKM120) post-drug addition, while for SOX2 immunoblotting, at 4 d post-drug addition. (D) Schematic for the shPIK3CA experiments. Cell colors are as described in (B). (E) Summary of shPIK3CA data. Means ± SEM of three replicates are shown. Significance was calculated using paired two-tailed *t* tests. *p = 0.01, **p = 0.003 (*SOX2*), 0.002 (*SPRR1A*), ***p = 0.0004 (*PIK3CA*), 0.0001 (*TMPRSS11B*), 0.00001 (*IVL*), 0.0002 (*SPRR2A*). All plotted numerical data are in S1 Data.

To determine if PI3K signaling is necessary for the squamous response to SOX2, we treated proliferating basal cell cultures on plastic with pan-class I/II/III and class I-specific PI3K inhibitors LY294002 and BKM120, respectively [85,86], concurrently with Lenti-SOX2 infection. Due to growth suppression by these drugs (Fig 5A), assays were limited to short-term plastic cultures. Both drugs inhibited AKT activation and suppressed squamous, but not mucinous, differentiation, without affecting Lenti-SOX2 expression (Fig 5B and 5C). Notably, in some experiments, the class I (p110α, β, δ, γ)-specific drug, BKM120, enhanced *MUC16* expression. We corroborated these findings with shRNA knockdown of *PIK3CA*, which is in the 3q amplicon. shPIK3CA reduced SOX2-dependent squamous differentiation and enhanced *MUC16* expression (Fig 5E), suggesting p110α may not only promote squamous differentiation but may also dampen the mucinous response to SOX2.

**Fig 6:**
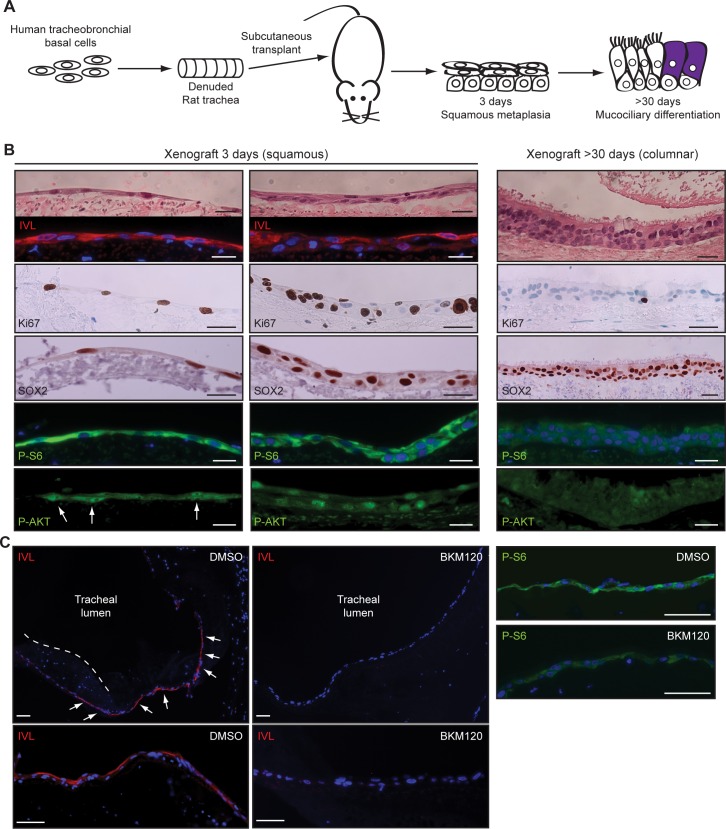
PI3K is necessary for squamous metaplasia in rat tracheal xenografts. (A) Schematic for rat tracheal xenograft procedure. Human tracheobronchial basal cells were seeded into denuded rat tracheas, which were then implanted subcutaneously into immunocompromised mice. (B) Staining for Ki-67, SOX2, phospho-Ser240/244-S6 (P-S6), and phospho-Thr308-AKT (P-AKT) during the initial phase of squamous metaplasia (3 d) and later period of mucociliary differentiation (>30 d). (C) Basal cells were seeded ± 5 μM BKM120 into denuded rat tracheas, and epithelia were immunostained after 1 d. Representative images from duplicate experiments are shown. Arrows point to areas of squamous differentiation, as evidenced by involucrin (IVL) expression. Dotted line indicates underlying tracheal tissue that moved into the lumen during sectioning. Scale bars are 20 μm (B) and 50 μm (C).

To determine if PI3K is necessary for squamous metaplasia in vivo, we seeded basal cells into denuded rat tracheas and engrafted them into immunocompromised mice (Fig 6A). In this setting, basal cells undergo squamous differentiation before regenerating a mucociliary epithelium [20], which can be recognized by expression of squamous markers such as IVL (Fig 6B). This response was associated with high Ki-67 and SOX2 expression and PI3K signaling (Fig 6B). Consistent with the in vitro data, when basal cells were seeded with BKM120, even though PI3K signaling was only partially inhibited, IVL expression was suppressed (Fig 6C). Finally, additional support for involvement of PI3K signaling in the squamous injury response of basal cells in vivo, as well as its importance to smoking-induced squamous lesions, comes from a phase I clinical trial with *myo*-inositol [87]. In this trial, *myo*-inositol, a natural precursor to second messengers in the phosphatidylinositol cycle, enhanced regression of low-grade squamous dysplasias in current and former smokers while reducing PI3K signaling [74,87,88].

### SOX2 and PI3K Signaling Cooperatively Repress Expression of *SOX9*, a Gene that Is Expressed Inversely to SOX2 during Mucociliary and Squamous Differentiation

**Fig 7:**
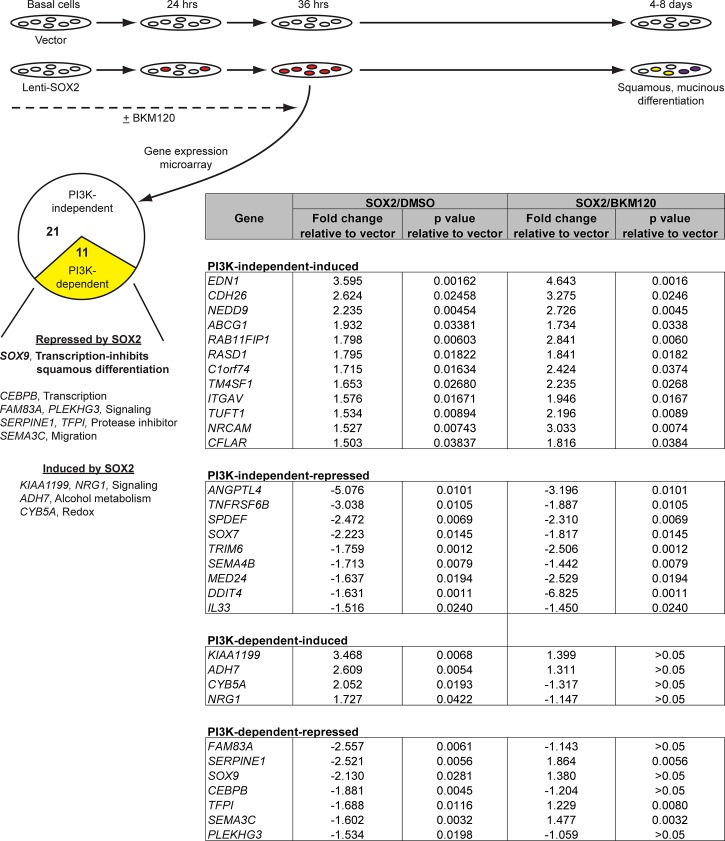
SOX2 and PI3K coregulate gene expression in tracheobronchial basal cells. Tracheobronchial basal cells growing on plastic were infected with Lenti-SOX2-FLAG or control vector ± 2.5 μM BKM120. After 36 hr, when most basal cells express high levels of SOX2 protein (S4B Fig), gene expression was analyzed by microarrays. In the schematic, white cells represent undifferentiated basal cells. Yellow and purple depict squamous and mucinous-differentiating cells, respectively. Red denotes cells that have been transduced with Lenti-SOX2. Results are shown from triplicate experiments.

To characterize the mechanism by which SOX2 induces squamous metaplasia from stem cells, we searched for basal cell target genes whose regulation by SOX2 is PI3K-dependent. We first established a time window during which stem cells might be undergoing SOX2-dependent changes that affect lineage determination. For these experiments, we used a FLAG-tagged SOX2 construct that retained the ability to induce mucinous and squamous differentiation (S4A Fig). By 36 hr post-Lenti-SOX2 infection of plastic basal cell cultures, most cells expressed SOX2 protein, as well as the basal cell marker TP63, but did not yet express markers of overt mucinous or squamous differentiation (S4B and S4C Fig). We therefore used 36 hr as an early time point to identify functionally important genes. SOX2 targets were identified by comparing gene expression between Lenti-SOX2 and vector-transduced cultures, with PI3K-dependency being established with Lenti-SOX2/BKM120 co-treatment. Using this strategy, we identified 32 SOX2-dependent changes in gene expression (1.5-fold or more), with 11 being PI3K-dependent and some targets being induced while others were repressed (Fig 7).

**Fig 8:**
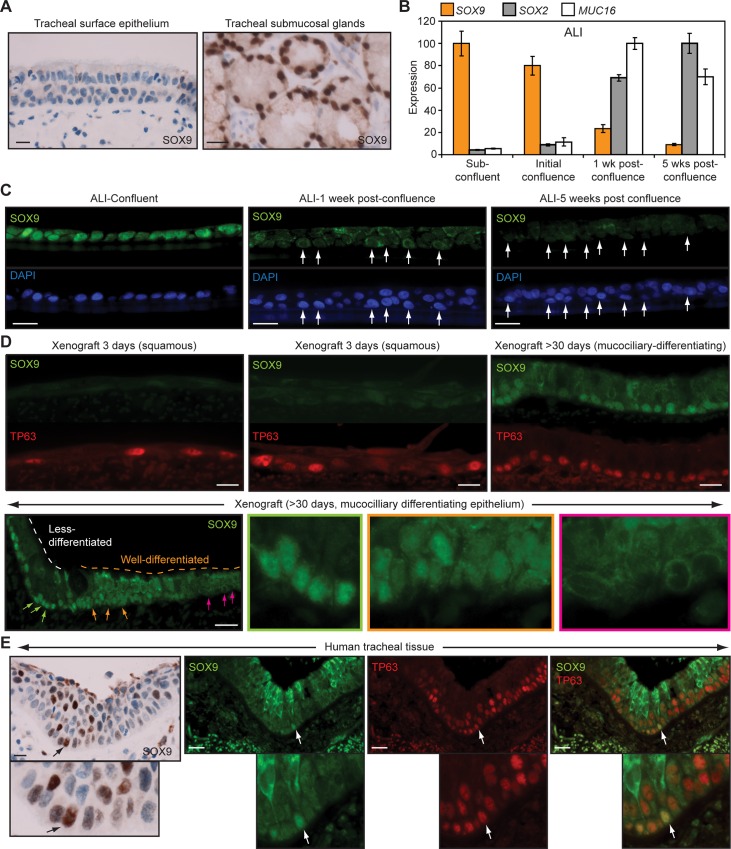
*SOX9* is a repressed target of SOX2 and PI3K that is expressed oppositely to SOX2. (A) SOX9 protein expression is not detected in the surface epithelium of native human tracheal tissue, but is observed in submucosal glands. (B, C) Analysis of SOX9 expression during mucociliary differentiation of tracheobronchial basal cell ALI cultures. (B) qRT-PCR quantification of *SOX9* mRNA expression. Data are plotted relative to the time point with maximal expression, which was given a value of 100. Means ± SEM from three replicates are shown. Plotted numerical data are in S1 Data. (C) SOX9 immunostaining. Arrows show positions of some basal cells. (D) Immunostaining of SOX9 expression in rat tracheal xenografts that have reconstituted a human tracheal epithelium. Grafts were generated as described in Fig 6A. Arrows point to basal cells in different epithelial regions that appear less (green) or more (orange, pink) columnar-differentiated and have differing amounts of SOX9 expression. Magnified images from these areas are shown and are bordered with colors matching the arrow colors. (E) SOX9 expression is occasionally detected in TP63-expressing basal cells in non-fully mucociliary differentiated human tracheal surface epithelia. Arrows point to basal cells with high SOX9 expression. All scale bars are 20 μm.

*SOX9* was one target that was repressed by SOX2 only when BKM120 was omitted from the culture media, which we confirmed by qRT-PCR (S5A Fig). In the absence of Lenti-SOX2, BKM120 treatment was sufficient to increase *SOX9* expression (S5B Fig), suggesting that PI3K signaling might act parallel to SOX2 in repressing *SOX9* expression. However, we did identify a SOX2 binding site in the *SOX9* promoter region (S5C Fig), supporting *SOX9* being a direct target of SOX2. Because ectopic SOX9 expression was reported to inhibit squamous differentiation of esophageal and skin basal cells [89,90], its natural expression in tracheobronchial basal cells might be a way of preventing squamous differentiation when mucociliary epithelia are required. In native human tracheal mucociliary epithelia, SOX9 expression was not detected, although it was expressed in submucosal glands (Fig 8A). Since the proliferative phase of ALI culture identifies a transient state when stem cells are initially committing to mucociliary fates, and transcription factors such as SOX2 show a different expression pattern relative to the differentiated quiescent state, we examined SOX9 expression in ALI cultures. In these cultures, SOX9 was expressed oppositely to SOX2 (Fig 8B and 8C versus Fig 1C and 1D). Highest expression of SOX9 was in proliferating basal cells and declined during mucociliary differentiation, explaining its absence in native quiescent mucociliary epithelia.

In agreement with our data indicating that SOX2 and PI3K cooperatively repress *SOX9* expression, SOX9 was not detected in squamous metaplasias of rat tracheal xenografts, which had high SOX2 expression and PI3K signaling (Figs 8D and 6B, day 3). However, SOX9 was expressed later, during regeneration of mucociliary epithelia when PI3K signaling was not active (Figs 8D and 6B, >30 d). In these epithelia, SOX9 expression was higher in areas that appeared incompletely differentiated and was absent in the most well-differentiated areas (Fig 8D, compare green and pink insets). This observation is consistent with the ALI data that shows a temporal decline in SOX9 expression during mucociliary differentiation and suggests that the SOX9-positive areas in the xenograft epithelia are at an earlier stage of differentiation.

Given the transient association of SOX9 expression with early generation of mucociliary epithelia, we re-examined SOX9 expression in native human tracheobronchial epithelia, focusing on areas that might be newly formed. In agreement with our observations in ALI and xenograft epithelia, we identified some areas where TP63-positive basal cells retained nuclear SOX9 expression, which was otherwise generally excluded from nuclei (Fig 8E). Thus, SOX9 appears to be transiently expressed when basal cells are initially committing to mucociliary fates.

### Murine Tracheal Basal Cells Transiently Enter a SOX2^Lo^SOX9^Hi^ State during Regeneration of Mucociliary Epithelia following Injury

**Fig 9:**
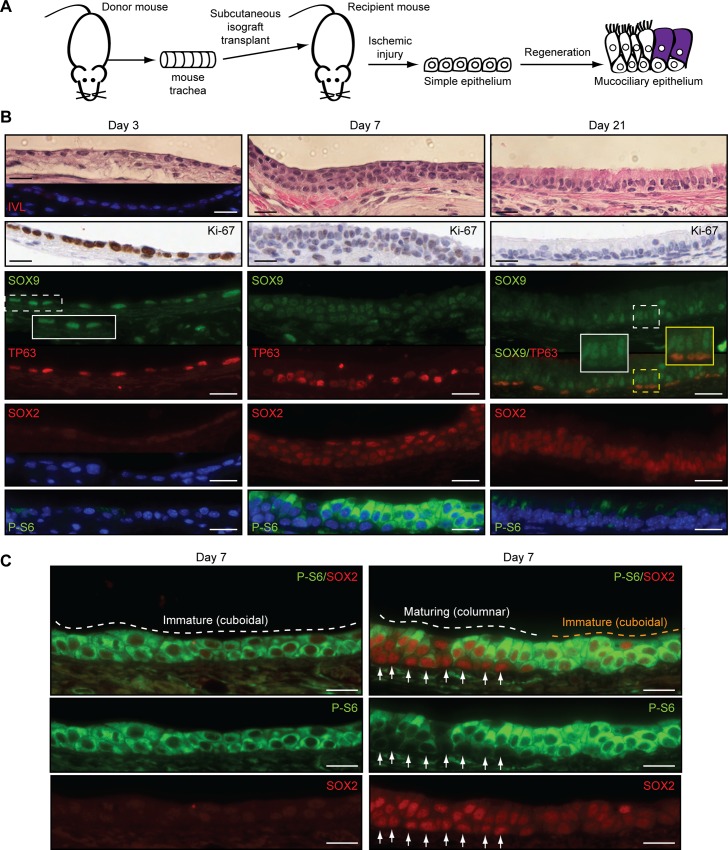
Murine tracheal basal cells transiently enter a SOX2^Lo^SOX9^Hi^ state during regeneration of mucociliary epithelia. (A) Murine tracheas were excised from donor mice and transplanted subcutaneously into recipient mice. (B) Following the initial ischemic injury, marker expression was examined by immunostaining at the indicated time points during tracheal epithelial regeneration. Insets show magnified areas denoted by the dashed boxes. Note that the SOX9-positive cells in the mature columnar-differentiated epithelium are not basal cells. (C) Maximal SOX2 expression and PI3K activity are mutually exclusive during regeneration of the columnar epithelium in murine tracheal isografts. Isograft epithelia from day 7 of regeneration were immunostained with the indicated antibodies. Left panel shows an immature cuboidal epithelium with uniformly high P-S6 and low SOX2 expression. Right panel shows an area transitioning from cuboidal to columnar epithelium. Arrows point to the basal cells underneath the columnar cells that have gained SOX2 expression and have lost much of the P-S6 signal. P-S6 = α-phospho-Ser240/244-S6. Scale bars are 20 μm.

To better capture a potential transient SOX2^Lo^SOX9^Hi^ state that precedes mucociliary differentiation in vivo, we used a murine tracheal isograft model of epithelial regeneration (Fig 9A). When murine tracheas are subcutaneously isografted into recipient mice, ischemic damage is repaired without histologic signs of squamous differentiation or IVL expression (day 3 epithelia) (Fig 9B). During the early proliferative phase of repair (day 3), basal cells expressed uniformly high SOX9 but low levels of SOX2 and PI3K activity (Fig 9B). Later, after 30 d, SOX2 expression increased throughout the columnar-differentiating epithelium, while SOX9 levels declined in basal cells and became restricted to some columnar cells (see insets), which could be secretory club cells that are not found in the human tracheobronchial epithelium. PI3K was transiently activated, but peaked in basal cells when SOX2 levels were low and declined as SOX2 expression rose (Fig 9C). Thus, both human and murine tracheobronchial basal cells appear to transiently enter a SOX2^Lo^SOX9^Hi^ state prior to generation of mucociliary epithelia.

### *SOX9* Promotes Basal Cell Growth and Its Repression Is Necessary for SOX2 to Induce Squamous Metaplasia

**Fig 10:**
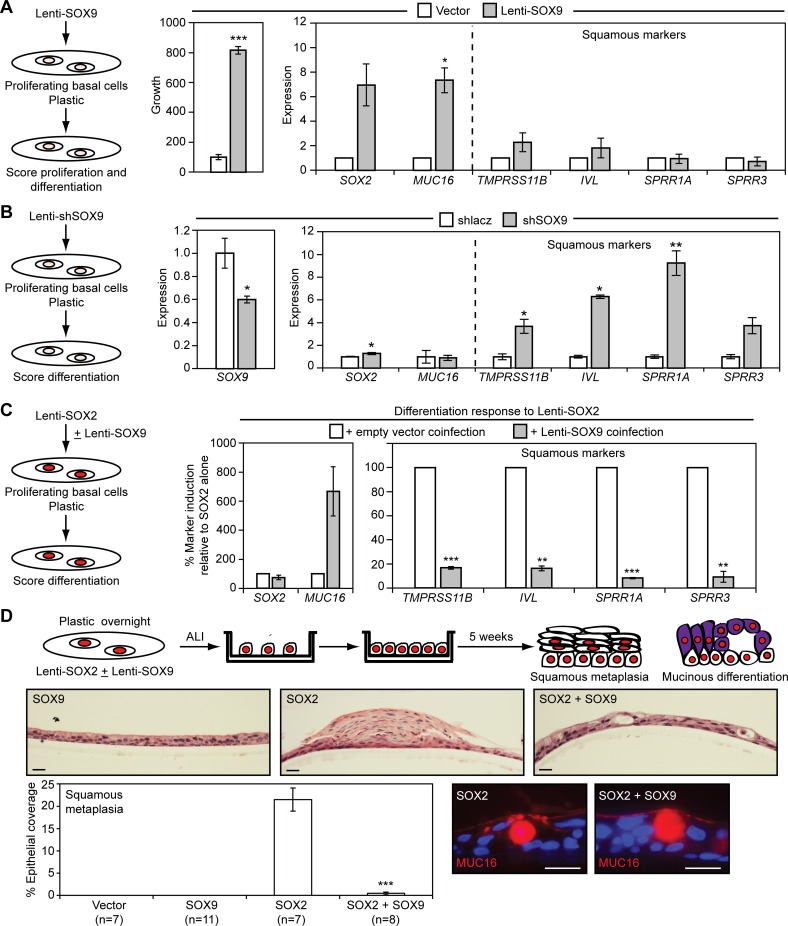
SOX9 promotes basal cell proliferation and inhibits squamous differentiation. (A) Elevation of SOX9 expression in tracheobronchial basal cells promotes growth and induces *MUC16* expression. Basal cells proliferating on plastic were infected with Lenti-SOX9 or control vector. The red color in all of the schematics indicates Lenti-SOX2-transduced cells. For growth assays, 5 d after infection, cells were replated at low density and cell number quantified after 7 d by alamarBlue. Data are normalized to the amount of growth in control vector cultures, which was given a value of 100. Means ± SEM from quadruplicate cultures are shown. Significance was calculated using a two-tailed *t* test. ***p = 0.0000005. For lineage marker expression analysis, mRNA was isolated 5 d following Lenti-SOX9 infection and quantified by qRT-PCR. Data are normalized to expression in control vector cultures, which was given a value of 1. Means ± SEM of three replicates are shown. Significance was calculated using paired two-tailed *t* tests. *p = 0.02. (B) shSOX9 spontaneously increases expression of squamous markers in plastic cultures of tracheobronchial basal cells. Basal cells growing on plastic were infected with shlacz or shSOX9; after 5 d, lineage marker expression was measured by qRT-PCR. Data are plotted relative to expression in shlacz control cultures, which was given a value of 1. Means ± SEM from three replicates are shown. Significance was calculated using two-tailed *t* tests. *p = 0.05 (*SOX9*), 0.03 (*SOX2*), 0.02 (*TMPRSS11B*), 0.01 (*IVL*), 0.003 (*SPRR1A*). (C) Constitutive SOX9 expression suppresses SOX2-induced squamous differentiation in plastic cultures of tracheobronchial basal cells. Basal cells were infected with empty vector, Lenti-SOX2 alone, or Lenti-SOX2 + Lenti-SOX9; after 5 d, lineage marker expression was measured by qRT-PCR and analyzed as described in Fig 5B. Fold inductions were first calculated by comparing marker expression between Lenti-SOX2 and control vector (non-SOX2)-transduced cells. The fold-inductions were then compared between matched pairs of Lenti-SOX2 and Lenti-SOX2 + Lenti-SOX9 coinfected cultures. The amount of marker induction in coinfected cultures was then plotted as a percentage of the induction observed with Lenti-SOX2 alone, which was given a value of 100. Means ± SEM of three replicates are shown. Significance was calculated using paired two-tailed *t* tests. **p = 0.0006 (*IVL*), 0.002 (*SPRR3*), ***p = 0.0002 (*TMPRSS11B*), 0.00004 (*SPRR1A*). (D) Constitutive SOX9 expression suppresses SOX2-induced histologic squamous metaplasia in tracheobronchial basal cell cultures grown at ALI. Basal cells were infected with control vector or Lenti-SOX2 ± Lenti-SOX9 and grown at ALI for 5 wk. Squamous differentiation was quantified by scoring 10 cm of epithelium per replicate. Significance was calculated using a two-tailed *t* test relative to Lenti-SOX2 alone. ***p = 0.000001. MUC16 expression was not affected by Lenti-SOX9 coinfection with Lenti-SOX2, as assessed by immunostaining. Scale bars are 20 μm. All plotted numerical data are in S1 Data.

Given that SOX9 expression is highest during the earliest stages of mucociliary differentiation, SOX9 may affect pre-differentiation properties of stem cells such as proliferation and lineage commitment. To determine how SOX9 affects the stem cell behavior of tracheobronchial basal cells, we first used a constitutively expressing lentiviral vector to raise its expression in plastic cultures of SOX2^Lo^ basal cells. Lenti-SOX9 increased basal cell growth while elevating expression of *SOX2* and *MUC16*, but not markers of squamous differentiation (Fig 10A). The increase in *SOX2* mRNA expression did not translate to a detectable increase in SOX2 protein levels (S6 Fig), and the *MUC16* induction was generally weaker than observed with Lenti-SOX2. These data suggest that SOX9 may have a role in promoting columnar lineage commitment, which is associated with a limited ability to increase *MUC16* mRNA expression. Analysis of the effects of reduced SOX9 expression was problematic, as *SOX9* shRNA lentiviruses suppressed basal cell growth. However, in short-term plastic cultures, partial *SOX9* knockdown increased squamous marker, but not *MUC16* expression (Fig 10B). The amount of squamous marker induction was also generally weaker than observed for Lenti-SOX2, supporting the concept that SOX9 is an early determinant of columnar versus squamous lineage commitment, rather than a strong inducer of differentiation.

Because *SOX9* expression is repressed by SOX2, we next determined the consequences of sustained SOX9 expression on SOX2-induced basal cell differentiation. When co-infected with Lenti-SOX2 in plastic cultures, Lenti-SOX9 suppressed squamous differentiation while enhancing *MUC16* expression (Fig 10C). Similarly, in ALI cultures, coinfection of Lenti-SOX9 with Lenti-SOX2 inhibited histologic squamous metaplasia, but not MUC16 differentiation (Fig 10D). Thus, *SOX9* repression is part of the mechanism through which SOX2 and PI3K promote squamous metaplasia from stem cells.

### SOX9 Is Not Expressed in High Grade Dysplasia when SOX2 Expression and PI3K Signaling Are High

**Fig 11:**
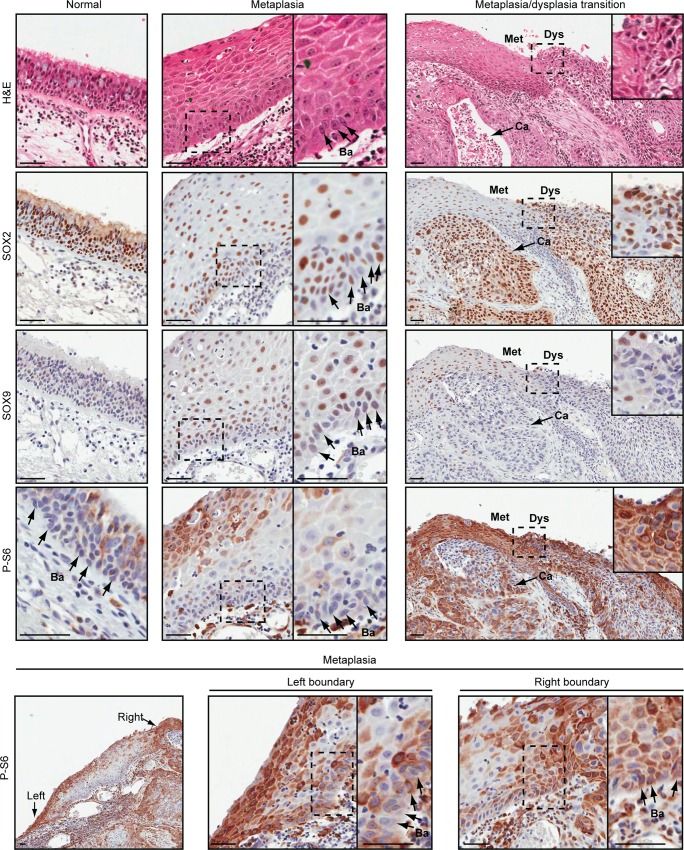
SOX2, SOX9, and phospho-S6 (P-S6) expression during SQCC pathogenesis. Stage 2A lung resection from a 45 packs/year smoker showing normal respiratory epithelium, squamous metaplasia, high grade dysplasia, and invasive squamous carcinoma. Met = squamous metaplasia, Dys = high grade dysplasia, Ca = carcinoma. Arrows point to representative basal cells (Ba). Insets show magnified areas within the dashed boxes. In squamous metaplasia (including basal cells), the percentage of P-S6-positive cells and the intensity of staining per cell increased towards areas of dysplasia. All scale bars are 50 μm.

To further investigate if the mechanism regulating squamous differentiation in stem cells might contribute to progression through early stages of SQCC pathogenesis, we characterized SOX2, SOX9, and P-S6 expression during preneoplasia (Fig 11). We analyzed an SQCC resection from a smoker that included normal respiratory epithelium, squamous metaplasia, and high-grade dysplasia, in addition to frank carcinoma. As expected, most cells in the normal mucociliary epithelium expressed SOX2, while SOX9 was not detected, and P-S6 expression was confined to some columnar, but not basal cells (Fig 11). In squamous metaplasia, SOX2 was expressed in most cells, but heterogeneously in the basal and suprabasal layers. The heterogeneity in basal cells would be consistent with ongoing squamous differentiation of some basal cells (SOX2^Hi^ cells), while other basal cells are poised to regenerate columnar cells during later regression (SOX2^Lo^ cells). SOX9 was not expressed in most basal cells of squamous metaplasia, which supports these cells either being squamous-committed or in an early stage of transitioning to columnar fates. Surprisingly, however, SOX9 was heterogeneously expressed in suprabasal cells, possibly signifying a non-stem cell function for SOX9 in some squamous differentiated progeny (see also S7 Fig). P-S6 staining was intense in the uppermost layers of squamous metaplasia but varied in the basal cell compartment. In the centermost basal region, P-S6 staining was low, while its intensity increased in basal cells as they approached areas of dysplasia (lowermost panels). By contrast, in high-grade dysplasia, expression of SOX2, SOX9, and P-S6 was uniform. SOX2 and P-S6 were expressed at high levels, while SOX9 expression was lost (see also S7 Fig), supporting most dysplastic cells being stem cell-like and committed to the squamous fate. The dysplastic expression patterns were maintained in the invasive carcinoma (S7 Fig).

### SOX9 Is Generally Expressed at Low Levels in SQCCs, with Highest Expression Associated with Different Clinical and Molecular Phenotypes

We next characterized SOX2, SOX9, and P-S6 expression in a panel of 132 SQCCs using tissue microarrays. First, we evaluated SOX2 and SOX9 expression by IHC using an H-score. Because the basal-most cells could be more stem cell-like and, hence, better recapitulate expression patterns we observed during normal stem cell differentiation, basal and suprabasal layers were scored independently. Overall, 94% of SQCCs expressed SOX2, with most tumor cells expressing moderate to high levels, and 73% had detectable levels of SOX9, although in the majority of cases SOX9 was expressed in very few tumor cells and at low levels (S8A and S8B Fig, Table 1). While SOX2 and SOX9 expression were expected to be inversely correlated, we did not observe a strong negative correlation between their H-scores (S8C Fig). We also expected that SOX9 levels might vary inversely to the extent of squamous differentiation. Although there was a tendency for histologically well-differentiated tumors to have lower SOX9 and higher SOX2 H-scores (Table 1), this trend was not significant. Similarly, when focusing on the highest quartile of SOX9-expressing tumors, there was no difference in differentiation, as compared to the 75% of cases with lower SOX9 expression (S8D Fig). Given that SOX2 and SOX9 H-scores were not that different between basal and suprabasal layers, for P-S6, we only scored the fraction of tumor cells that stained positive. In 92% of SQCCs, some P-S6 staining was detectable, and 91% of SQCCs that expressed SOX2 also expressed P-S6, although in many cases, few tumor cells expressed P-S6 (S9A and S9B Fig). As with SOX2 and SOX9, we did not detect strong negative correlations between P-S6 and SOX9 expression (S9C Fig). However, the narrow spreads in the majority of SOX2, SOX9, and P-S6 expression data may have limited the power of our statistical analyses, and histological tumor grade might not have been sensitive enough to detect differences in the extent of squamous differentiation. Nevertheless, the generally high and low expression of SOX2 and SOX9, respectively, are consistent with SOX2 inhibiting SOX9 expression and SOX9 potentially interfering with squamous differentiation in SQCCs.

**Table 1:** Characterization of SOX2 and SOX9 expression in primary patient SQCCs.

Tumor differentiation	H-score^a^				RNAseq^b^	
	Basal		Suprabasal			
	SOX2	SOX9	SOX2	SOX9	SOX2	SOX9
Well	175 ± 20.5	33.7 ± 14.9	157 ± 21.0	31.3 ± 7.4	NA	NA
Moderate	147 ± 7.9	37.1 ± 6.2	149 ± 7.4	41.0 ± 5.8	NA	NA
Poor	142 ± 12.2	35.7 ± 10.4	143 ± 11.1	37.3 ± 10.1	NA	NA
Overall	149 ± 6.4	36. 4 ± 5.0	148 ± 5.9	39.1 ± 4.5	4,081 ± 289	906 ± 64

^a^H-score: The H-score was derived from SOX2 and SOX9 IHC of a tissue microarray constructed from a cohort of 132 SQCC patients. The H-score was calculated for each core by summing: [(0 x % cells with no stain) + (1 x % cells with weak stain) + (2 x % cells with moderate staining) + (3 x % cells with intense staining)]. The H-score scale ranged from 0 to 300. Basal and suprabasal layers were scored separately with the hypothesis that in moderate and well-differentiated tumors, stem cells might reside in the basal layers and more differentiated progeny would be found in suprabasal areas. Means ± SEM are shown. The individual patient tumor H-score data are in S2 Data.

^b^*SOX2* and *SOX9* mRNA expression data were obtained from the TCGA analysis of 177 primary patient SQCCs. Data are in RPKM (reads per kilobase of transcript per million mapped reads), with the means ± SEM shown. The TCGA patient data are in S2 Data.

**Fig 12:**
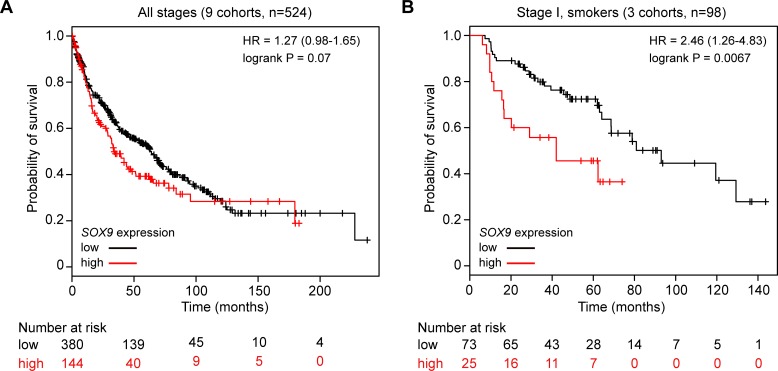
*SOX9* mRNA expression is associated with poor probability of survival for SQCC patients. Univariate Kaplan-Meier survival analysis of SQCC patients, stratified by *SOX9* mRNA expression. (A) Analysis using data pooled from nine cohorts and not filtered by grade or smoking history. (B) Analysis filtering on Stage I and smoking history (three cohorts). All plotted numerical data are in S1 Data.

As a potentially more sensitive way of characterizing *SOX2* and *SOX9* expression in SQCCs, we also examined their mRNA levels as quantified by RNAseq in the TCGA SQCC cohort [42]. Consistent with our IHC data, in most SQCCs, *SOX9* was expressed at much lower levels than *SOX2*, and there was a weak negative correlation between their expression (S10A and S10B Fig, Table 1). Although *SOX9* expression was generally low across SQCCs, given its ability to promote basal cell proliferation (Fig 10A), we hypothesized that highest expressors might have distinct characteristics. In support of this possibility, the upper quartile of cases, which, on average, had 4-fold higher *SOX9* expression than the remaining 75% of cases, were associated with fewer high copy number *SOX2* gains (amplification) (S10C Fig). Furthermore, when using publicly available data from nine SQCC cohorts and stratifying by approximately the upper quartile, there was a tendency for high *SOX9* expression to be associated with a lower probability of survival (Fig 12A). This association was significant when restricting analysis to patients with stage I disease and a smoking history (Fig 12B), suggesting that SOX9 might promote more aggressive behavior in early stage SQCCs.

Finally, we used gene set enrichment analysis to explore phenotypic differences between SQCCs that expressed low or high levels of *SOX9*. We first used the TCGA SQCC RNAseq data to characterize *SOX9-*low and -high expressors by compiling lists of the top 200 genes that were either most anti-correlated or correlated with *SOX9* expression (S1 and S2 Tables). We then used these lists to identify related cell lines in the Cancer Cell Line Encyclopedia (CCLE). Consistent with absence of SOX9 promoting squamous differentiation, genes anti-correlated with *SOX9* only showed significant associations with SQCC cell lines (S3 Table). Conversely, in agreement with SOX9 inhibiting squamous differentiation, genes correlated with *SOX9* did not show associations with any SQCC cells lines (S3 Table). However, genes correlated with *SOX9* did show relationships to cell lines derived from neural crest (melanoma, glioblastoma) and breast tumors, whose normal tissue and cancer origins are affected by SOX9 [91–99].

## Discussion

SQCCs have been grouped into four subtypes based on differences in gene expression, which have been proposed to arise through different mechanisms involving distinct cells of origin [42,100]. While at some point transformation mechanisms may diverge, most SQCCs likely originate in basal cells through their natural squamous injury response [20,25]. TP63 protein, a hallmark of basal cells, is expressed in virtually all SQCCs, with 96% of cases having strong expression [13–15], and it is generally accepted that most SQCCs arise following squamous metaplasia [101]. How the squamous injury response is initiated and exploited to promote SQCC pathogenesis has been a mystery.

Here, we show that SOX2 and PI3K cooperate to induce the squamous injury response in basal cells. Although in the normal tracheobronchial epithelium SOX2 is expressed at high levels in most basal cells, its expression can vary during injury and regeneration, with a SOX2^Lo^ state preceding new mucociliary differentiation. The SOX2^Lo^ state appears to facilitate ciliogenesis, while high levels of SOX2 induce distinct types of lineage differentiation, depending on the signaling context. SOX2 can induce a specific type of mucinous differentiation marked by MUC16, or it can drive basal cells into a hyperplastic state that differentiates into squamous epithelia. The squamous response specifically requires PI3K signaling, which cooperates with SOX2 to repress *SOX9*, an inhibitor of squamous differentiation in several stratifying epithelia [89,90]. In embryonic stem (ES) cells, AKT directly regulates SOX2 through phosphorylation at Thr116, which increases SOX2 stability and, thus, generally promotes its activity [78,79]. However, upon PI3K inhibition in basal cells, SOX2 protein expression is not reduced, and one differentiating activity of SOX2 (squamous) is much more affected than the other (mucinous). We also find that PI3K signaling inhibits *SOX9* expression even in the absence of Lenti-SOX2 transduction. Hence, PI3K may act parallel to SOX2, possibly through chromatin modifications, which can be modulated by PI3K signaling [102,103]. PI3K may also help establish the squamous fate as the dominant SOX2 response by antagonizing mucinous differentiation, since shPIK3CA enhances SOX2-dependent induction of *MUC16* expression.

Under uninjured conditions, turnover is low in the tracheobronchial epithelium [16]. Most basal cells express high levels of SOX2, while in rare cells, SOX2 expression is low. SOX2^Lo^ cells may be committed to the ciliated fate, or they may be in a naïve state that has not yet committed to a particular columnar lineage. By contrast, SOX2^Hi^ cells could be committed to a mucinous fate or a ciliated fate, if SOX2 expression increased after ciliated cell commitment. Further work will be required to address these questions as well as to determine at what point initial lineage commitments become irreversible. However, given that PI3K signaling is low in basal cells in the uninjured state, squamous differentiation should not occur.

Smoking is the major initiating factor for SQCC pathogenesis [17,18]. Cigarette smoke condensate induces squamous differentiation in vivo and in cultures of tracheobronchial basal cells [24,25]. Furthermore, cigarette smoke extract and nicotine increase AKT activation in tracheobronchial basal cells [72,73], and cytologically normal epithelia from smokers with dysplasia have elevated PI3K activity [74]. Thus, smoking likely triggers squamous metaplasia at least partly through PI3K activation in basal cells. Although SOX2^Lo^ basal cells should be able to adopt a squamous fate in response to PI3K activation, SOX2 expression would first have to increase, which is not yet known to be a response to smoking. Alternatively, if some of the many SOX2^Hi^ basal cells are not irreversibly committed to columnar fates, PI3K activation in these cells might be sufficient to trigger a rapid widespread squamous response. Ultimately, SOX2 is expressed in many basal cells of smoking-associated squamous metaplasia, supporting its role in driving metaplasia. However, SOX2, as well as P-S6, are heterogeneously expressed amongst these basal cells. Because functional PI3K activity may be below our detection limits in some cells, and because it is not certain where the stem cells in the metaplasia reside (e.g., center: low P-S6 versus edges: high P-S6, Fig 11), the significance of the P-S6 heterogeneity is not clear. On the other hand, regarding heterogeneity in SOX2 expression, it might be that SOX2^Hi^ basal cells are actively involved in ongoing squamous differentiation, while SOX2^Lo^ basal cells are poised to later regenerate mucociliary epithelia, as we observed during regeneration in various injury models. It is not clear why SOX9 is expressed in suprabasal metaplastic layers, but it may relate to an unknown function in squamous differentiated progeny. Importantly, because squamous metaplasia basal cells are hyperproliferative [21], the longer they remain in this state, the greater the risk of acquiring genetic driver events from genotoxic damage. Some of the hyperproliferation may be an intrinsic property of the squamous-committed state. In Lenti-SOX2 ALI cultures that contained adjacent squamous and non-squamous epithelia, only basal cells associated with squamous metaplasia were hyperproliferative, and in the absence of smoke.

**Fig 13:**
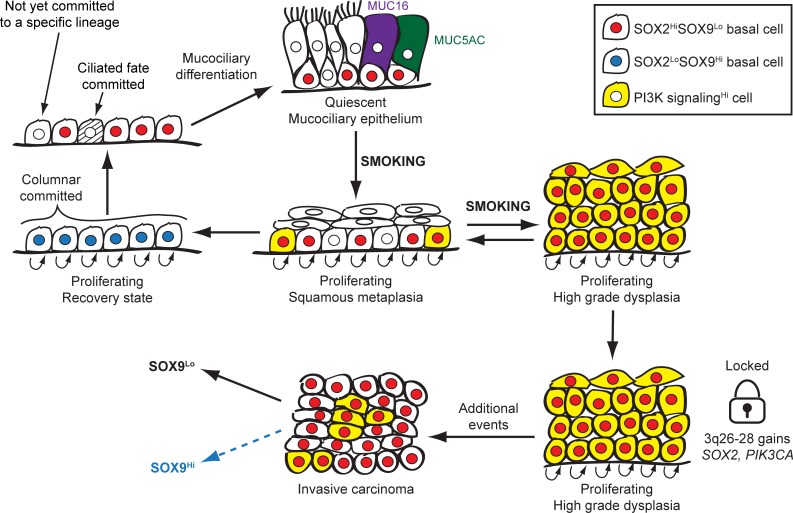
Stem cell model for the role of *SOX2* and *PIK3CA* coamplification in SQCC pathogenesis. SOX2 (red nuclei), SOX9 (blue nuclei), and PI3K signaling (yellow cytoplasm) levels are highlighted in basal cells of normal and metaplastic epithelia and in all cells of dysplasia and SQCC. In basal cells of uninjured mucociliary epithelia, SOX9 and PI3K signaling levels are low, and most, but not all, cells express SOX2. Smoking induces PI3K activation, which may cause SOX2^Hi^ basal cells that have not irreversibly committed to mucociliary fates to enter a hyperproliferative squamous metaplastic state. Alternatively, PI3K signaling may trigger an increase in SOX2 expression in SOX2^Lo^ basal cells, which also drives entry into the hyperproliferative squamous metaplastic state. In squamous metaplasia basal cells, SOX2 and PI3K signaling levels are heterogeneous. This heterogeneity may signify the presence of both squamous-committed basal cells, as well as basal cells that are poised to regenerate mucociliary epithelia once injury has subsided. With continued smoking, squamous metaplasia progresses to high-grade dysplasia, in which most cells are in a SOX2^Hi^SOX9^Lo^ state, with high levels of PI3K signaling. In this state, the majority of cells are hyperproliferative and committed to a squamous fate, although they do not undergo extensive differentiation. In non-3q-amplified dysplasia and metaplasia, the squamous injury state in basal cells subsides as SOX2 expression and PI3K signaling naturally decline over time. Basal cells then enter a proliferative SOX2^Lo^SOX9^Hi^ state, which promotes columnar over squamous fates. From this state, ciliated cell commitment occurs while SOX2 levels are low, and later, as SOX9 expression decreases and SOX2 levels increase, quiescence and mucociliary differentiation occur (with SOX2 inducing the MUC16 mucinous fate [purple]). By contrast, in high-grade dysplasias that have acquired copy number gains at 3q26-28, SOX2 expression and PI3K signaling are maintained and prevent the squamous injury response from waning. Eventually, additional genetic events accrue in clones such that transformation to invasive carcinoma occurs. SQCCs diverge in the level of PI3K signaling and SOX9 expression. While in most SQCCs, SOX9 is expressed at low levels, a subset of SQCCs have higher SOX9 expression, which inhibits the squamous phenotype and may drive more aggressive tumor behavior.

High-grade dysplasia is a turning point in SQCC pathogenesis in that it appears to mark a transition from an environmentally reactive pathology to one with a strong genetic component. It is the earliest stage when common focal genetic changes are detected [36], and it is less prone to regression than earlier stages [28,33,34]. During this stage, we find that SOX2 and P-S6 are highly expressed in most cells. These observations agree with previous reports that noted high levels of SOX2 and P-AKT in dysplasia [75–77,104]. The high expression levels could signify a largely stem cell-like population with strong commitment to the squamous fate. Because some high-grade dysplasias regress [29,32,33,105], the dysplastic phenotype is not likely to be strictly dependent on genetic alterations. We propose, however, that in order to regress, SOX2 and PI3K signaling levels must decline (Fig 13). This reduction would be hindered in dysplasias that acquire *SOX2* and *PIK3CA* coamplification. Such 3q-amplified dysplasias would be more prone to progression due to the sustained hyperproliferative squamous-committed stem cell state and the inability to regress to quiescent mucociliary differentiated epithelia. As such, we would predict that 3q amplification would be a biomarker for non-regressing dysplasias.

While in theory, as long as there is regular exposure to smoke, a squamous injury state should be sustainable without 3q amplification, this may not actually be the case. First, our finding that tracheobronchial basal cells in normal tissue express similar levels of SOX2 as *SOX2*-amplified SQCCs suggests that *SOX2* promoter activity may decline during SQCC pathogenesis. Second, premalignant squamous lesions regress in smokers [28–30], and, intriguingly, regression follows the reverse steps of progression [28,105], supporting waning of the squamous injury response over time. This is not surprising, as many cellular responses desensitize after chronic stimulation. Moreover, the squamous injury response involves the same stem cells that perpetually renew the epithelium. Hence, it would be important for stem cells to have a natural mechanism that limits the duration of the squamous injury response and exposure of replicating stem cells to genotoxic stress.

Targeting the squamous injury response as early as possible would minimize accrual of genetic drivers and, hence, potentially be chemopreventative. One approach could involve PI3K inhibition. Notably, *myo*-inositol, a natural sugar alcohol, enhanced regression of low-grade dysplasias in a phase I clinical trial [87]. Although *myo*-inositol can affect multiple signaling pathways [106], in the phase I trial, regression was accompanied with PI3K pathway inhibition [74,88], which our data support as being part of the mechanism of regression. Thus, natural and targeted PI3K inhibitors should continue to be investigated. We would also suggest that even dysplasias with 3q amplification might respond to anti-PI3K therapy. We and others have found that PI3K signaling is high in dysplasia [74–77], and our stem cell data suggest that 3q amplification is initially selected to maintain a waning squamous injury response, which together, support 3q-amplified dysplastic cells being dependent on PI3K signaling. However, such basal cells would be prone to returning to a squamous injury state if therapy was discontinued.

Growth of many cancers is dependent on mechanisms that specify developmental lineages (e.g., [107, 108]), including SQCCs being dependent on SOX2 [41,71,109]. Because most SQCCs express high levels of SOX2 and low levels of SOX9 and show evidence of PI3K activity in at least some tumor cells (which could be stem cells), the mechanism we uncovered in stem cells may continue to promote squamous identity in SQCCs. Accordingly, PI3K may be a vulnerability in invasive carcinoma. However, in a clinical trial involving SQCC patients, BKM120 was ineffective [110]. This could reflect a problem with the therapeutic index, in which case co-targeting additional components of the squamous injury response mechanism might improve PI3K inhibitor effectiveness. Alternatively, failure of BKM120 could indicate that many SQCCs have lost the dependency on PI3K that is likely prevalent during early stages of progression. Notably, as compared to dysplasia, which had uniformly high SOX2 and P-S6 expression, in 50% of SQCCs, P-S6 expression was observed in 10% or less of tumor cells. On the other hand, in 20% of cases, P-S6 was expressed in at least 70% or more of tumor cells. Further work will be necessary to assess the roles of SOX2, SOX9, and PI3K in maintaining the squamous identity of SQCCs and to determine whether sensitivity to PI3K inhibitors varies with P-S6 expression.

We also discovered that although SOX9 was generally expressed at low levels and in few tumor cells, its expression varied among SQCCs. SOX9^Hi^ SQCCs were associated with fewer high copy number *SOX2* gains and a lower probability of survival, suggesting that they represent a distinct SQCC subclass that may at least partly be driven by SOX9. First, by gene set enrichment analysis, these tumors have less of a squamous phenotype, which could reflect the anti-squamous differentiation properties of SOX9. Second, we also found that SOX9 drives proliferation of normal basal cells and that, in several injury models, basal cells enter a transient SOX2^Lo^SOX9^Hi^ proliferative state prior to mucociliary differentiation. Thus, during later stages of pathogenesis, in a subset of SQCCs, genetic or epigenetic alterations may be selected that promote this natural, SOX9^Hi^ non-squamous-committed stem cell state. This basal cell state may share phenotypic and functional similarities with states established by SOX9 in several other cellular contexts. For example, our gene set enrichment analysis found similarity between SOX9^Hi^ SQCCs and certain breast cancers (especially triple negative) and neural crest-derived tumors, whose genesis and malignant properties are affected by SOX9. SOX9 promotes the mammary stem cell state, is associated with poor prognosis in breast cancer, and is thought to be especially active in triple negative tumors, where it enhances the tumorigenic phenotype [93,95,99]. SOX9 also induces neural crest identity and promotes generation of downstream melanocyte and glial lineages [92,94,96,97]. In addition, SOX9 promotes malignant properties of melanoma and glioblastoma cell lines and is associated with lower rates of survival for both cancers [91,98]. Interestingly, SOX9^Hi^ SQCCs also had similarity to several small cell lung cancer (SCLC) cell lines. However, the role of SOX9 in SCLC has not been examined. Further work should be done to better characterize SQCCs expressing different levels of SOX9 and how they may have distinct clinical outcomes and sensitivities to different treatment strategies.

Basal cells are also stem cells in other stratifying epithelia, where they make similar decisions regarding squamous and non-squamous fates [111]. As in the lung, homeostatic decisions can be altered by injury to induce hyperproliferative metaplasias that increase cancer risk, especially in the bladder, cervix, and esophagus [112–117]. These decisions may analogously be driven by SOX2 and SOX9, with injury-induced changes in their expression causing metaplasia. For example, SOX9 inhibits squamous differentiation of esophageal basal cells [89], and during development, the esophageal epithelium transitions from a columnar epithelium that coexpresses SOX2 and SOX9 to a squamous epithelium where only SOX2 is expressed [118–120]. Injury induced by chronic acid reflux reactivates SOX9 expression [119], and triggers Barrett’s esophagus, a gastric/intestinal metaplasia that increases the risk of developing adenocarcinoma in the formerly squamous epithelium [114]. Conversely, copy number gains at 3q are associated with SQCCs in different tissues (see also TCGA data, www.cbioportal.org) [41,121,122]. In some cases, such as in the lung and cervix, gains at 3q may be used to stabilize a more proliferative squamous metaplastic injury state in stem cells that do not normally exclusively generate a squamous epithelium. However, in other cases such as in the esophagus, where stem cells are already squamous-committed and quite proliferative [112], there is evidence that coamplification of *SOX2* and *PIK3CA* stabilize a non-metaplastic injury state that increases squamous stem cell self-renewal [123–125]. It thus appears that early steps of carcinogenesis in stratifying epithelia may generally involve environment-induced activation of stem cell injury states, with subsequent selection of genetic driver events that stabilize these states.

## Materials and Methods

### Ethics Statement

Normal human tracheobronchial (carinal) tissue and primary lung SQCC tissue were obtained with written informed consent from patients and with approval of the University Health Network Research Ethics Board (08-0318-T for normal tracheal tissue; 04-0557-T and 10-0158T for SQCC tissue). All animal work was carried out with the approval of the University Health Network Animal Care Committee, was registered and licensed under the province of Ontario’s Animals for Research Act, and was compliant with the humane policies and guidelines of the Canadian Council on Animal Care. Rat tracheas were obtained from deceased rats following CO_2_ asphyxiation using AUP1556. Primary patient SQCC tissue and rat tracheal xenografts were implanted into NOD/SCID mice that had been anesthetized with ketamine, following AUPs 603 and 1557, respectively. For murine tracheal isograft experiments, tracheas were harvested from donor mice following CO_2_ asphyxiation, and were subcutaneously transplanted into recipient mice that had been anesthetized with ketamine, following AUP 3480. At the experimental endpoints, mice were sacrificed by CO_2_ asphyxiation.

### Primary Human Tracheal Basal Cells

Normal human tracheobronchial (carinal) tissue was obtained as discard from lung transplant operations. Basal cells were isolated from tracheobronchial tissue as described [126] and expanded in LHC-9 medium [127] on Petri dishes coated with 48 μg/ml PureCol (Advanced BioMatrix). For subculturing, cells were treated with 0.025% trypsin/EDTA, followed by Trypsin Neutralizing Solution (Lonza). For air-liquid-interface (ALI) growth, 10,000 cells/well were seeded onto 0.4 μm 12 mm polyester Transwell-Clear membranes (Corning) coated with PureCol. Both chambers were initially incubated with LHC basal: DMEM (1:1) that was supplemented as described [59], which included 0.33 nM retinoic acid and 5 ng/ml EGF. Cultures typically took 7–10 d to reach confluence. After confluence, cells were only fed basolaterally, with retinoic acid and EGF concentrations changed to 50 nM and 0.5 ng/ml, respectively, to induce differentiation. ALI cultures were typically maintained for an additional 4–5 wk once the basolateral feeding regimen was begun. Cultures were grown at 37°C in 5% CO_2_, with media changed every other day. Cells were not expanded beyond two passages. The data presented in the manuscript were derived from multiple strains of basal cells that were isolated from more than 20 different donor carinas.

### SQCC Tissue Microarrays

Construction of the SQCC tissue microarrays (TMAs) was previously described [128]. The demographics and clinical characteristics of the SQCC cohort are summarized in S4 Table.

### Animal Work

Primary patient SQCC tissue was implanted subcutaneously into NOD/SCID mice as described [129]. Rat tracheal xenograft experiments involving isolation of rat tracheas and their denudation, seeding with human tracheobronchial basal cells, and subsequent implantation into immunocompromised mice, were performed as described [130]. Briefly, rat tracheas were harvested from Wistar male rats (226–250 g) after CO_2_ asphyxiation. Tracheas were denuded by three rounds of freeze/thaw and were seeded with 7.5 x 10^5^ human tracheobronchial basal cells. Seeded tracheas were then implanted subcutaneously into NOD/SCID male mice (7–8 wk old, ~18 g) that had been anesthetized with ketamine. For short-term experiments involving the PI3K inhibitor BKM120, rat tracheas were closed at both ends with surgical sutures rather than assembling them into cassettes as described in [130]. For murine tracheal isograft experiments, Balb/c mice (7–9 wk, 18 g) were used. Tracheas were excised from donor mice after CO_2_ asphyxiation, flushed with saline, and transplanted subcutaneously into recipient mice that had been anesthetized with ketamine. At the experimental endpoints, mice were sacrificed by CO_2_ asphyxiation.

### Genomic Profiling of Primary Patient-Derived SQCC Xenografts (PDXs)

Xenograft genomic copy number was measured on the HumanOmni 2.5 Beadchip SNP array platform (Illumina). The total signal intensity (LogR) and B-allele frequency (BAF) values were reported at each genomic locus that was profiled by the SNP array. Relative copy number gain or loss of genomic regions was identified using the ASCAT segmentation algorithm [131] with the assumption that the reference genome is diploid. We mapped each gene to a genomic region if more than 50% of the gene overlaps with the region. The average log likelihood ratio (LRR) for each gene was determined, which quantifies the relative copy number gain in the tumor. An LRR of 0.5 was considered as at least one copy number gain.

For exome sequencing, exomes were captured with the Agilent SureSelect Human 50Mbp kit and subjected to paired-end sequencing using the Illumina HiSeq platform. Xenome [132] was used to remove contaminating reads from the mouse stroma, and basic alignment and sequence quality control were done using Novoalign v3 and Picard v1.78. Mapped exomes were then processed by the standard GATK pipeline to perform additional quality control, variant calling, and mutational significance analysis. Somatic mutation calling with matched normal samples was also performed using Strelka v1.0 [133]. Mutations called by both GATK and Strelka were validated using targeted exome capture with the Agilent SureSelect Custom 2Mbp kit and the same pipeline. High confidence variants in PDXs included only mutations called by both exome and targeted sequence analysis or called in only exome sequence analysis but also found in the COSMIC database v70.

### FACS Analysis of Normal Human Tracheobronchial Basal Cell Cultures

First passage tracheobronchial cells were removed from plastic with 0.025% trypsin, incubated with trypsin neutralization solution (Lonza), pelleted, and resuspended in HBSS/2% FBS. 5 x 10^5^ cells were stained on ice for 30 min in 50 μL HBSS/2% FBS with APC-conjugated α-CD44 (eBioscience, 17-0441-81, 1:200). Cells were then washed three times in HBSS/2% FBS and analyzed.

### Tracheobronchial Basal Cell Growth Assays

Basal cells were seeded in triplicate at 2200 cell/cm^2^ into 12-well dishes (~15% confluence). Media was changed every other day and experiments terminated when control wells had reached ~80% confluence (typically 7–10 d). At the end point, cell growth was quantified by alamarBlue (ThermoFisher), as per the manufacturer’s instructions.

### Antibody Staining

Human tracheobronchial tissue, rat tracheal xenograft tissue, and ALI cultures were fixed in 10% buffered formalin, soaked in 70% ethanol, and then paraffin embedded. ALI filters were embedded in 3% agar prior to paraffin embedding. All immunohistochemistry was performed using the Vantana Benchmark XT autostainer with the iVIEW DAB detection kit (Vantana Medical Systems).

For immunofluorescence staining of tissue, sections were deparaffinized through successive incubations in xylene, and decreasing concentrations of ethanol and antigens were retrieved in 10 mM citrate buffer, pH 6.0, using the 2100 Retriever (Aptum Biologics, Ltd.). For immunofluorescence staining of tracheobronchial basal cells growing in plastic cultures, cells were cytospun onto charged glass slides and fixed in 4% paraformaldehyde. After fixation and antigen retrieval (for tissue sections), cells were permeabilized with 0.1% Triton X-100/PBS, blocked with 3% bovine serum albumin (BSA)/ 0.1% Triton X-100/PBS, and incubated with primary antibody diluted in 3% BSA/ 0.1% Triton X-100/PBS overnight at 4°C. Cells were then washed three times with 0.1% Triton X-100/PBS and incubated with secondary antibody in 3% BSA/ 0.1% Triton X-100/PBS for 2 hours at room temperature. Secondary antibodies were Alexafluor 488 goat anti-rabbit (Invitrogen, 1:500) and Alexafluor 568 goat anti-mouse (Invitrogen, 1:500). After secondary antibody staining, cells were washed three times with 0.1% Triton X-100/PBS and mounted in Vectashield mounting media containing DAPI (Vector Laboratories). For *en face* α-BTUB4 staining, after antibody staining, cells were dehydrated through a series of washes in increasing concentrations of ethanol (70%–100%). Filters were then cut from the Transwell insert and were mounted. For quantification of ciliogenesis by *en face* BTUB4 staining, images were taken on an Olympus IX81 microscope using a Rolera MGi Plus camera (QImaging) and were tiled and quantified using MetaMorph software (Molecular Devices). Primary antibodies for immunofluorescence staining were α-BTUB4 (#T7941, Sigma, 1:100), α-IVL (#ab68, Abcam, 1:1000), α-KRT5 (#ab24647, Abcam, 1:100), α-MUC5AC (Santa Cruz, #sc-20118, 1:100), α-MUC16 (#ab693, Abcam 1:100), α-SOX2 (#MAB2018, R&D systems, 1:50), α-TMPRSS11B (#HPA042951, Sigma, 1:600), α-TP63 (#sc-56188, BC4A4, Santa Cruz, 1:100), α-phospho-S6 (Ser240/244, #5364, Cell Signaling, 1:800), α-KRT5 (ab24647, Abcam, 1:1000), α-phospho-AKT (Thr308) (#2965, Cell Signaling, 1:200). Primary antibodies for immunohistochemistry were α-Ki-67 (#MIB-1, Dako, 1:150), α-HMWCK (#34BE12, Dako, 1:100), α- SOX9 (#AB5535, Millipore, 1:1,200), and α-SOX2 (#AF2018, R&D Systems, 1:2,000).

### Western Blotting

Whole-cell lysates were prepared in lysis buffer (1% SDS, 10% glycerol, 80 mM Tris-HCl, pH 6.8) that had been pre-heated to 95°C. Proteins were transferred to Immobilon-FL PVDF membranes (Millipore) in transfer buffer (50 mM Tris base, 40 mM glycine, 0.04% SDS, 10% methanol) with an Owl semidry transfer apparatus (Thermo Scientific). Membranes were incubated with primary antibodies overnight at 4°C in 3% skim milk in Tris-Buffered Saline Tween-20 (TBST) (50 mM Tris–HCl, 150 mM NaCl, 0.05% Tween-20 [pH = 8.0]). Primary antibodies were α-phospho-AKT (Thr308) (#2965, Cell Signaling, 1:1000), α-phospho-AKT (Ser473) (#4060, Cell Signaling, 1:1000), α-AKT (#2920, Cell Signaling, 1:1000), α-phospho-S6 (Ser240/244) (#5364, Cell Signaling, 1:1000), α-S6 (#2317, Cell Signaling, 1:1000), α-SOX2 (MAB2018, R&D systems, 1:500), and α-SHP2 (C-18, Santa Cruz, 1:2000). After primary antibody binding, membranes were washed three times with TBST and probed with anti-rabbit (IRDye 800RS, LI-COR Biosciences, 1:10,000) and goat anti-mouse (Alexa Fluor 680, Invitrogen, 1:15,000) secondary antibodies in TBST for 1 hour at room temperature. After washing three times in TBST, proteins were visualized using an Odyssey Infrared Imaging System (LI-COR Biosciences).

### Chemical Inhibitors

BKM120 was purchased from Selleckchem and LY294002 from Sigma.

### Plasmids

A full-length human *SOX2* cDNA in the pOTB7 cloning vector was purchased from the TCAG Genome Resource Facility (The Hospital for Sick Children). This cDNA was used to make two untagged expression constructs in the pMA1 lentiviral vector [134], which were indistinguishable in their biological activity. In this vector, a minimal CMV promoter drives GFP expression, while the PGK promoter drives constitutive expression of the gene of interest. pLenti-SOX2G was constructed by digesting pOTB7-SOX2 with EcoRI, blunting the 5′ end of *SOX2* with Klenow, then releasing *SOX2* by XhoI digestion and cloning the fragment into SmaI/SalI-digested pMA1. pLenti-SOX2B was constructed by cloning *SOX2* into pMALB, a variant of pMA1 in which GFP was replaced with TagBFP, and pMA1 was converted into a Gateway destination vector [135]. For this construct, *SOX2* was first cloned into the Gateway entry vector, pCR8/GW/TOPO (Life Technologies), after PCR amplification from pOTB7-SOX2 with SOX2-2 (5′-ctag tctaga cat gtg tga gag ggg cag tgt g-3′) and SOX2-6 (5′-actg gaat tca cat gtg tga gag ggg cag tg-3′). *SOX2* was then transferred to pMALB from pCR8 by Gateway Cloning (Life Technologies).

pLenti-SOX2-FLAG was constructed through a series of steps beginning with the PCR-based TA-cloning of *SOX2* into pGem-T-easy (Promega) using pOTB7-SOX2 as a template and the primers SOX2-1 (5’- Ccg gaattc ggc atg tac aac atg atg gag acg gag-3’) and SOX2-2 (5’-ctag tctaga cat gtg tga gag ggg cag tgt g-3’). An in-frame 3x FLAG tag was then added to the C-terminus of SOX2 by cloning *SOX2* from pGemSOX2 into p3xFLAG-CMV-14 (Sigma) via EcoRI and XbaI digestion and ligation. The FLAG-tagged *SOX2* cDNA was then amplified using primers CMVFLAG14-1 (5’-5’-TCCAG AGA TCT AGA GCT CGT TTA GTG AAC CGT CAG-3’) and CMVFLAG14-2 (5’-ATAGTA GAT CTG GGG AGG GGT CAC AGG GAT GCC-3’) and cloned into the Gateway entry vector, pCR8/GW/TOPO. SOX2-FLAG was then transferred to pMALB from pCR8 by Gateway cloning.

pLenti-SOX9 were constructed by Gateway cloning of the human SOX9 cDNA from a Gateway entry vector (GeneCopoeia) into pMAL, a pMA1 derivative that was modified into a Gateway destination vector that still coexpresses GFP [135].

### Lentiviral Infection

VSV-G-pseudotyped lentiviruses were generated by cotransfection of lentiviral vector and standard packaging plasmids into 293T cells by calcium phosphate. At 48 and 72 hr post-transfection, viruses were concentrated with Lenti-X (Clontech), resuspended in LHC-9, and stored at -80°C. For infections, basal cells were seeded at 2,100 cells/cm^2^ on plastic and 36 hours post-seeding, infected in 8 μg/ml polybrene. Cells were infected overnight and either maintained on plastic or seeded after 36 hours into ALI culture. Generally, ~90% transduction was achieved. Puromycin was used at 3 μg/ml to select for shRNA lentiviruses. Lentiviral packaging plasmids included pCMVΔR 8.91, pRSV-Rev, and pMD.G [136]. Lentiviral shRNA constructs were obtained from Open Biosystems and included shPIK3CA (TRCN0000196795), shSOX9 (V3LHS_396211), shluc (TRCN0000072246), and shlacz (TRCN0000072235).

### RNA Analysis

RNA was isolated using a Micro RNA kit (Ambion) followed by DNAse I treatment. cDNA was prepared using a High Capacity cDNA Reverse Transcriptase kit (ABS), and qRT-PCR was performed with either SYBR green (Bio-Rad) or Taqman probes (ABI). Gene expression was quantified by the ΔΔCt method with TBP normalization [137]. Primers are listed in S5 Table.

### Microarrays

Three biological replicate experiments were set up, with each one consisting of an empty vector and two Lenti-SOX2-FLAG-transduced conditions. At the time of virus addition, the empty vector and one of the Lenti-SOX2 conditions were treated with 0.1% DMSO, while the other Lenti-SOX2 condition was treated with 2.5 μM BKM120. After overnight virus incubation, cells were washed in Hepes-buffered saline and refed with LHC-9 containing either BKM120 or DMSO. Thirty-six hours post virus addition, RNA was harvested. The Illumina TotalPrep-96 RNA Amplification Kit was used to generate biotinylated, amplified cRNA for hybridization with Illumina Human HT-12 v4.0 Expression BeadChips. Quantile normalized data were analyzed in R and Genespring (Agilent). Genes significantly changed by SOX2/DMSO or SOX2/BKM120 were identified by comparing the replicates of each condition with the control vector/DMSO replicates using a one-way ANOVA test and a Benjamini-Hochberg-corrected *p* < 0.05 cutoff and a post-hoc Tukey’s HSD test. Genes that were significantly changed by SOX2 in the same direction regardless of BKM120 treatment were classified as PI3K-independent. Genes that were significantly changed with SOX2/DMSO, but were not significantly changed or were significantly changed in the opposite direction by BKM120 treatment, were classified as PI3K-dependent. A minimum 1.5-fold change in response to SOX2/DMSO was used as another cut-off. Data have been deposited to GEO (http://www.ncbi.nlm.nih.gov/geo/) with the accession number GSE59866.

### TCGA Data

Provisional lung SQCC TCGA data were used and were downloaded from www.cbioportal.org [138].

### Gene Set Enrichment Analysis

The top 200 *SOX9* correlated or anti-correlated genes (S1 and S2 Tables) were searched for enrichment of gene sets annotated for cell lines contained in the CCLE, using the Enrichr tool (http://amp.pharm.mssm.edu/Enrichr/) [139,140]. Statistical significance of the enrichment was assessed by a “q-value,” which is a *p*-value that has been adjusted using the Benjamini-Hochberg method for correction for multiple hypotheses testing. Only cell lines yielding q-values ≤ 0.05 were deemed to have enrichment of the test genes.

### Kaplan-Meier Survival Analysis

The data used in Fig 12A are derived from nine lung SQCC cohorts. Eight cohort datasets can be downloaded from GEO (http://www.ncbi.nlm.nih.gov/geo/) with the accession numbers GSE14814, GSE19188, GSE29013, GSE30219, GSE3141, GSE37745, GSE4573, and GSE50081. The ninth cohort is from the TCGA and can be downloaded from the NIH Genomic Data Commons https://gdc.cancer.gov/. The data used in Fig 12B are derived from three lung SQCC cohorts, which can be downloaded from GEO with the accession numbers GSE4573, GSE50081, and GSE29013. All of the data were mined through the web tool Kaplan-Meier Plotter (http://kmplot.com/analysis/) [141]. Both analyses used the *SOX9* 202935_s_at Affymetrix probe, and the “auto-select” mode was used to determine the optimal cut-off for high and low *SOX9* expression, which was approximately the upper quartile. The numerical data used to generate the plots are in S1 Data.

## Supporting Information

S1 Data Data values plotted in main figures. (XLSX)

S2 Data Data values plotted in supplemental figures. (XLSX)

S3 Data CD44.fcs file for FACS analysis shown in S2B Fig. (FCS)

S4 Data Unstained control.fcs file for FACS analysis shown in S2B Fig. (FCS)

S5 Data Flowjo.jo file for CD44 FACS analysis shown in S2B Fig. (JO)

S1 Fig Characterization of SOX2 expression in native human tracheobronchial epithelia and SQCCs. (A) Box plot analysis of TCGA *SOX2* RNAseq data from 177 primary patient SQCCs. Numerical data are in S2 Data. RPKM = Reads Per Kilobase of transcript per Million mapped reads. (B) SOX2 IHC in native human tracheobronchial epithelia and *SOX2*-amplified primary patient lung SQCC xenografts (PDXs). Arrows point to some basal cells. Note the abundance of cilia in the normal tracheal epithelium and hence, high SOX2 expression in ciliated cells. (C) SOX2^Lo^ basal cells are rare in well-differentiated columnar epithelia, but are more common in non-columnar epithelia. Native human tracheal tissue was costained with α-SOX2 and α-KRT5 antibodies. Arrows point to rare SOX2^Lo^ basal cells in well-differentiated columnar epithelia. Insets are magnified areas marked by dashed line boxes. All scale bars are 20 μm.
(TIF)

S2 Fig Characterization of normal human tracheobronchial basal cells. (A, B) Basal cell lineage marker expression in primary P0 tracheobronchial cell cultures growing on plastic. Data are from one representative strain. (A) Immunostaining for TP63 and KRT5 expression. (B) FACS analysis of CD44 expression. FACS data files are available as S3 Data, S4 Data, and S5 Data. (C) Evidence for retention of multipotent stem cell activity by tracheobronchial basal cells growing on plastic. Passage 2 basal cells were transplanted into denuded rat tracheas, which were implanted subcutaneously into immunocompromised mice, and examined histologically after 55 days. Regenerated mucociliary surface epithelia and submucosal glands are highlighted with dashed lines. Data are from one representative strain. Scale bars are 20 μm (A) and 50 μm (C).
(TIF)

S3 Fig PI3K signaling is low in basal cells in columnar differentiated native human tracheal epithelia. Tracheal tissue was stained for phospho-Ser240/244-S6 (P-S6) or phospho-Thr308-AKT (P-AKT). Insets correspond to magnified areas bounded by dashed boxes. White and black arrows point to representative basal cells (Ba). Representative columnar cells (Co) with nuclear P-AKT are indicated by red arrows. Scale bars are 20 μm.
(TIF)

S4 Fig Characterization of SOX2-FLAG activity and kinetics of lineage marker expression after Lenti-SOX2-FLAG transduction of tracheobronchial basal cells. (A–C) Tracheobronchial basal cells growing on plastic were infected with control vector or Lenti-SOX2-FLAG and analyzed as indicated. (A) SOX2-FLAG induces markers of mucinous and squamous differentiation in basal cells. Five days following Lenti-SOX2-FLAG transduction, lineage marker expression was measured by qRT-PCR. Data are plotted relative to uninfected controls, which were assigned a value of 1 and generally had the same baseline marker expression as empty-vector infected cells. Means ± standard error of the mean (SEM) from three replicates are shown. Significance was calculated using paired two-tailed *t* tests. * *p* = 0.008 (*MUC16*), 0.04 (*TMPRSS11B*), 0.01 (*IVL*), 0.05 (*SPRR1A*). (B) Kinetic analysis of SOX2 and TP63 protein expression after Lenti-SOX2-FLAG transduction. Positive cells were identified by immunofluorescence staining, with 150–250 cells counted. (C) Time course of lineage marker induction following Lenti-SOX2-FLAG transduction. Marker expression was quantified by qRT-PCR. Data are plotted relative to the time point with the greatest amount of marker expression, which was assigned a value of 100. All plotted numerical data are in S2 Data.
(TIF)

S5 Fig SOX9 expression is repressed by SOX2 and PI3K signaling. (A) Tracheobronchial basal cells growing on plastic were infected with control vector or Lenti-SOX2-FLAG ± 2.5 μM BKM120, and *SOX9* levels quantified by qRT-PCR 36 hours post-infection. Data were normalized to expression in vector-transduced cells, which was assigned a value of 100. Means ± SEM from four replicates are shown. Significance was calculated using paired two-tailed *t* tests. **p = 0.003. (B) Tracheobronchial basal cells growing on plastic were treated with control DMSO vehicle or 2.5 μM BKM120. After 3 d, *SOX9* expression was analyzed by qRT-PCR. Data were normalized to expression in DMSO-treated cultures, which was assigned a value of 1. Means ± SEM from three replicates are shown. Significance was calculated using a paired two-tailed *t* test. *p = 0.05. (C) Analysis of the human *SOX9* promoter for SOX2 binding sites. 3,000 bp of genomic sequence upstream of the first *SOX9* exon was scanned for the SOX2 motif MA0143.3 using the search tool at the JASPAR database (http://jaspar.genereg.net). A strong match was identified approximately 1,200 bp upstream of the first exon. All plotted numerical data are in S2 Data.
(TIF)

S6 Fig Lenti-SOX9 does not induce SOX2 protein expression in tracheobronchial basal cells. Basal cells proliferating on plastic were infected with control vector or Lenti-SOX9, and after 5 d, SOX2 expression was examined by immunoblotting 30 μg of lysate. The H520 SQCC cell line was used as a positive control.
(TIF)

S7 Fig Additional characterization of SOX2, SOX9, and phospho-S6 (P-S6) expression in SQCC preneoplasia and invasive disease. Larger areas of preneoplasia and representative areas of invasive disease from the lung resection shown in Fig 11. Sections were stained with the indicated antibodies. P-S6 = phospho-Ser240/244-S6. Dotted lines denote basolateral boundaries of metaplasia (Met) and dysplasia (Dys). Scale bars are 50 μm.
(TIF)

S8 Fig Characterization of SOX2 and SOX9 protein expression in SQCCs. (A–C) IHC for SOX2 and SOX9 expression in a tissue microarray (TMA) derived from an SQCC cohort of 132 patients. (A) Representative SOX2 and SOX9 IHC in the TMA. Scale bars are 50 μm. (B) Distribution of SOX2 and SOX9 H-scores in the SQCC cohort. Data were derived from the TMA and each patient core was given an H-score for SOX2 and SOX9 expression (see also Table 1). The H-score was calculated for each core by summing: [(0 x % cells with no stain) + (1 x % cells with weak stain) + (2 x % cells with moderate staining) + (3 x % cells with strong staining)]. The H-score scale thus ranged from 0–300. Basal and suprabasal layers were scored separately with the hypothesis that in moderate and well-differentiated tumors, stem cells might reside in the basal layers and more differentiated progeny would be found in suprabasal areas. (C) Relationship between SOX2 and SOX9 protein expression in SQCC patients. (D) Comparison of number of cases by tumor grade in high versus low SOX9-expressors. All plotted numerical data are in S2 Data.
(TIF)

S9 Fig Characterization of phospho-Ser240/244-S6 expression in SQCCs. (A) Representative images of P-S6 IHC in a tissue microarray (TMA) derived from an SQCC cohort of 132 patients (same as S8 Fig). Scale bar is 50 μm. (B) Distribution of P-S6 expression data in the SQCC cohort. (C) Relationship between P-S6 and SOX9 expression in SQCC patients. All plotted numerical data are in S2 Data.
(TIF)

S10 Fig Characterization of *SOX2* and *SOX9* mRNA expression in SQCCs. (A–C) All mRNA expression and copy number variation data are from the TCGA analysis of 177 primary SQCCs and are in S2 Data. (A) Distribution of mRNA expression across the patient cohort. RPKM = Reads Per Kilobase of transcript per Million mapped reads. (B) Relationship between *SOX2* and *SOX9* mRNA expression in SQCC patients. (C) Comparison of *SOX9*-high versus *SOX9*-low SQCCs and their associations with *SOX2* amplification. Statistical significance was calculated using a two-tailed Fisher’s exact test.
(TIF)

S1 Table Top 200 genes anti-correlated with *SOX9* mRNA expression in SQCCs. Data were obtained from the TCGA analysis of 177 primary patient SQCCs. Genes are ranked by the negative Pearson correlation coefficients.
(XLSX)

S2 Table Top 200 genes correlated with *SOX9* mRNA expression in SQCCs. Data were obtained from the TCGA analysis of 177 primary patient SQCCs. Genes are ranked by the positive Pearson correlation coefficients.
(XLSX)

S3 Table Identification of cell lines with associations to genes either anti-correlated or correlated with *SOX9* expression in SQCCs. The top 200 *SOX9* anti-correlated or correlated genes (S1 and S2 Tables) were searched for enrichment in gene sets annotated for cell lines contained in the CCLE (Cancer Cell Line Encyclopedia), using the Enrichr tool (http://amp.pharm.mssm.edu/Enrichr/). ^a^ Statistical significance of the enrichment, as assessed by a calculated “q-value,” which is a *p*-value that has been adjusted using the Benjamini-Hochberg method for correction for multiple hypotheses testing. Only cell lines yielding q-values ≤ 0.05 are shown. ^b^ SCLC = small cell lung cancer.
(XLSX)

S4 Table Demographics and clinical characteristics of the SQCC cohort used in the tissue microarray studies. (XLSX)

S5 Table Primers used for qRT-PCR. (XLSX)

## Abbreviations

ADC: adenocarcinoma
ALI: air-liquid-interface
CCLE: Cancer Cell Line Encyclopedia
ES cells: embryoninc stem cells
H&E: hematoxylin and eosin
HMWCK: high molecular weight cytokeratin
IHC: immunohistochemistry
IVL: involucrin
PAS: periodic acid-Schiff
PAS-D: periodic acid-Schiff-diastase
PDX: patient-derived xenograft
qRT-PCR: quantitative reverse transcription-polymerase chain reaction
SCLC: small cell lung cancer
SEM: standard error of the mean
SPRR: small proline-rich proteins
SQCC: squamous cell carcinoma
TCGA: The Cancer Genome Atlas
