# Supplementary figures and images for: SOX2 and PI3K Cooperate to Induce and Stabilize a Squamous-Committed Stem Cell Injury State during Lung Squamous Cell Carcinoma Pathogenesis
Source: PLoS Biol. 2016 Nov 23;14(11):e1002581. doi: 10.1371/journal.pbio.1002581 (PMC5120804; doi:10.1371/journal.pbio.1002581)

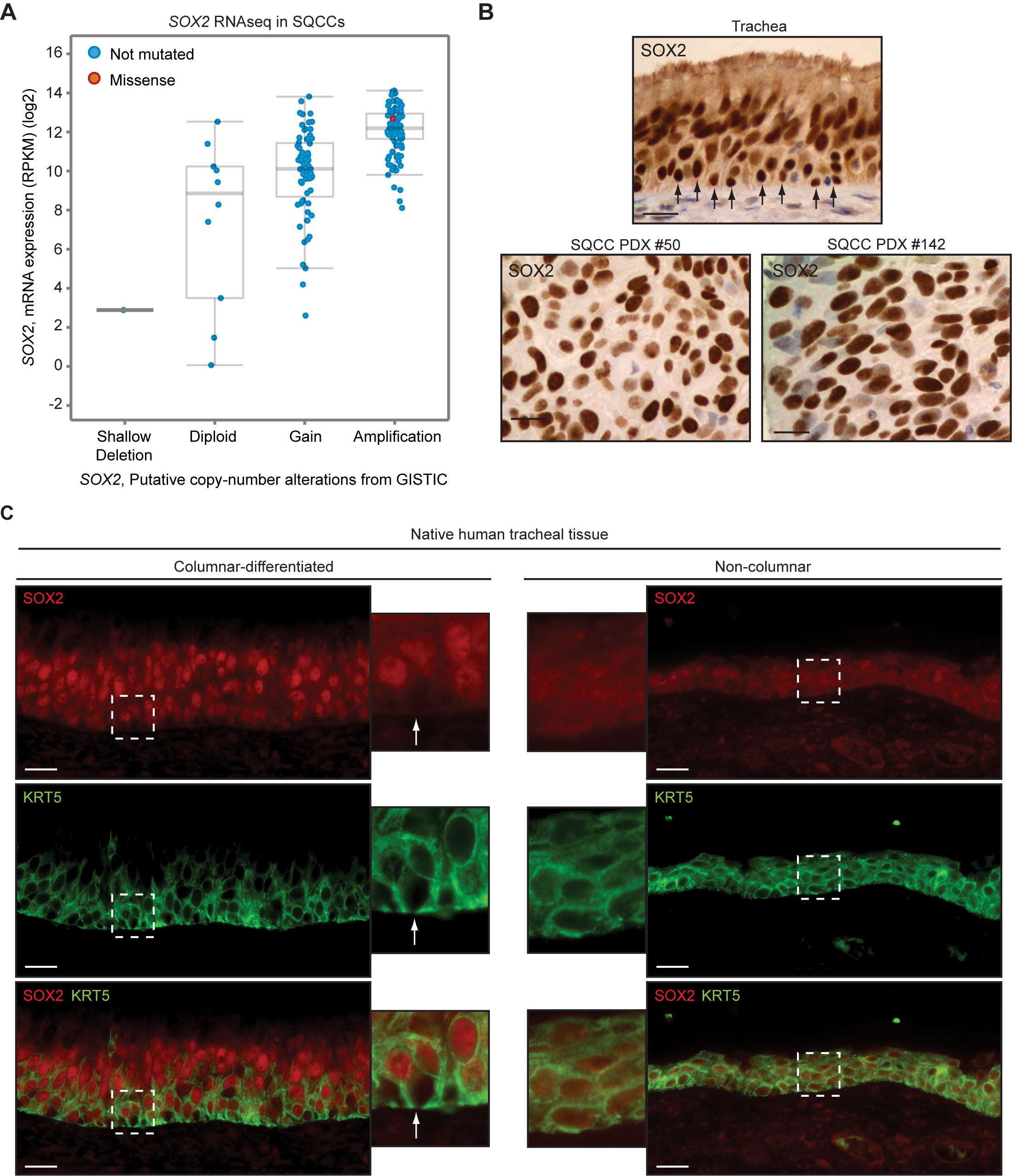

Supplement: S1 Fig — (A) Box plot analysis of TCGA SOX2 RNAseq data from 177 primary patient SQCCs. Numerical data are in S2 Data. RPKM = Reads Per Kilobase of transcript per Million mapped reads. (B) SOX2 IHC in native human tracheobronchial epithelia and SOX2-amplified primary patient lung SQCC xenografts (PDXs). Arrows point to some basal cells. Note the abundance of cilia in the normal tracheal epithelium and hence, high SOX2 expression in ciliated cells. (C) SOX2Lo basal cells are rare in well-differentiated columnar epithelia, but are more common in non-columnar epithelia. Native human tracheal tissue was costained with α-SOX2 and α-KRT5 antibodies. Arrows point to rare SOX2Lo basal cells in well-differentiated columnar epithelia. Insets are magnified areas marked by dashed line boxes. All scale bars are 20 μm. (TIF) [file pbio.1002581.s006.tif]

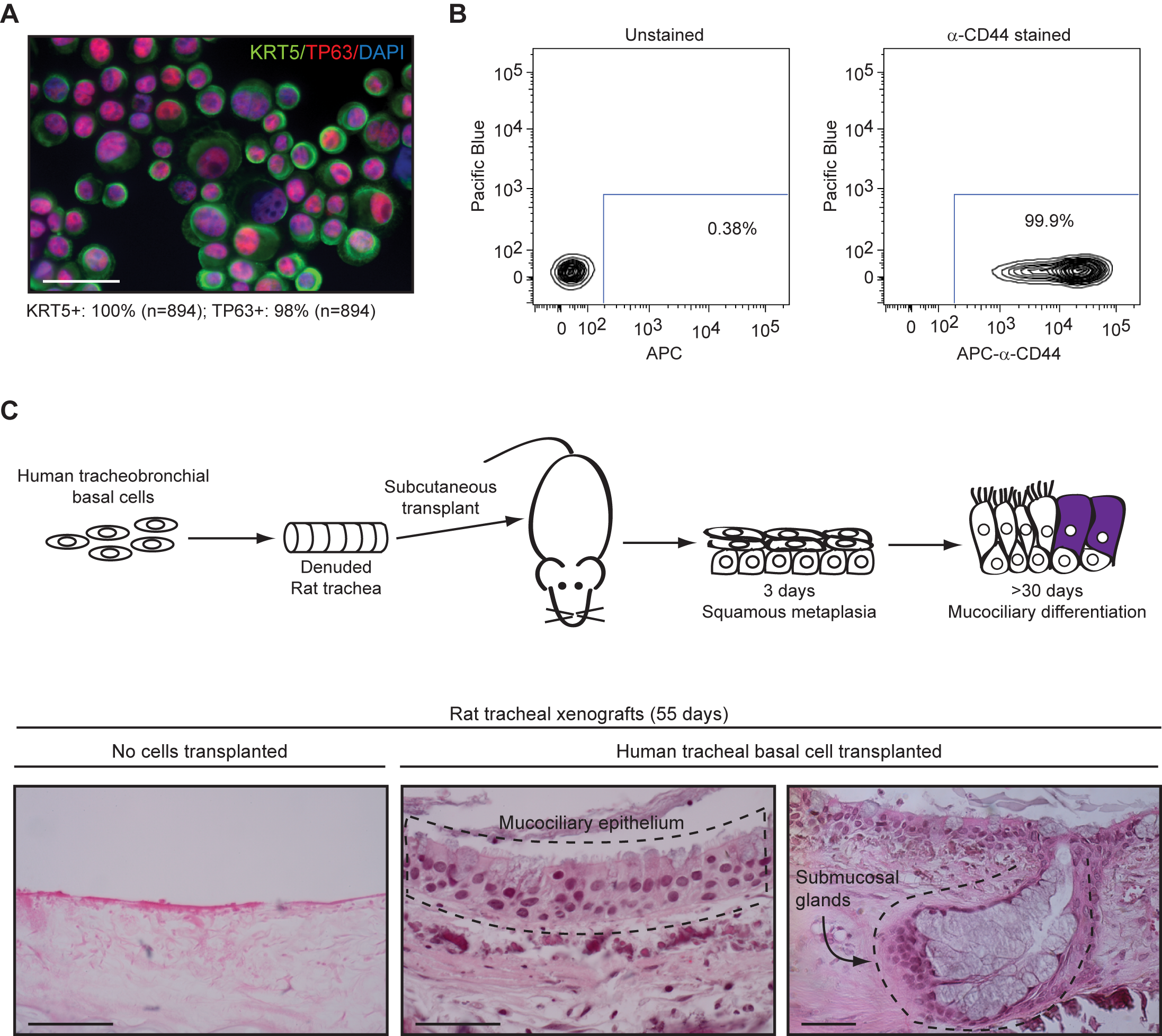

Supplement: S2 Fig — (A, B) Basal cell lineage marker expression in primary P0 tracheobronchial cell cultures growing on plastic. Data are from one representative strain. (A) Immunostaining for TP63 and KRT5 expression. (B) FACS analysis of CD44 expression. FACS data files are available as S3 Data, S4 Data, and S5 Data. (C) Evidence for retention of multipotent stem cell activity by tracheobronchial basal cells growing on plastic. Passage 2 basal cells were transplanted into denuded rat tracheas, which were implanted subcutaneously into immunocompromised mice, and examined histologically after 55 days. Regenerated mucociliary surface epithelia and submucosal glands are highlighted with dashed lines. Data are from one representative strain. Scale bars are 20 μm (A) and 50 μm (C). (TIF) [file pbio.1002581.s007.tif]

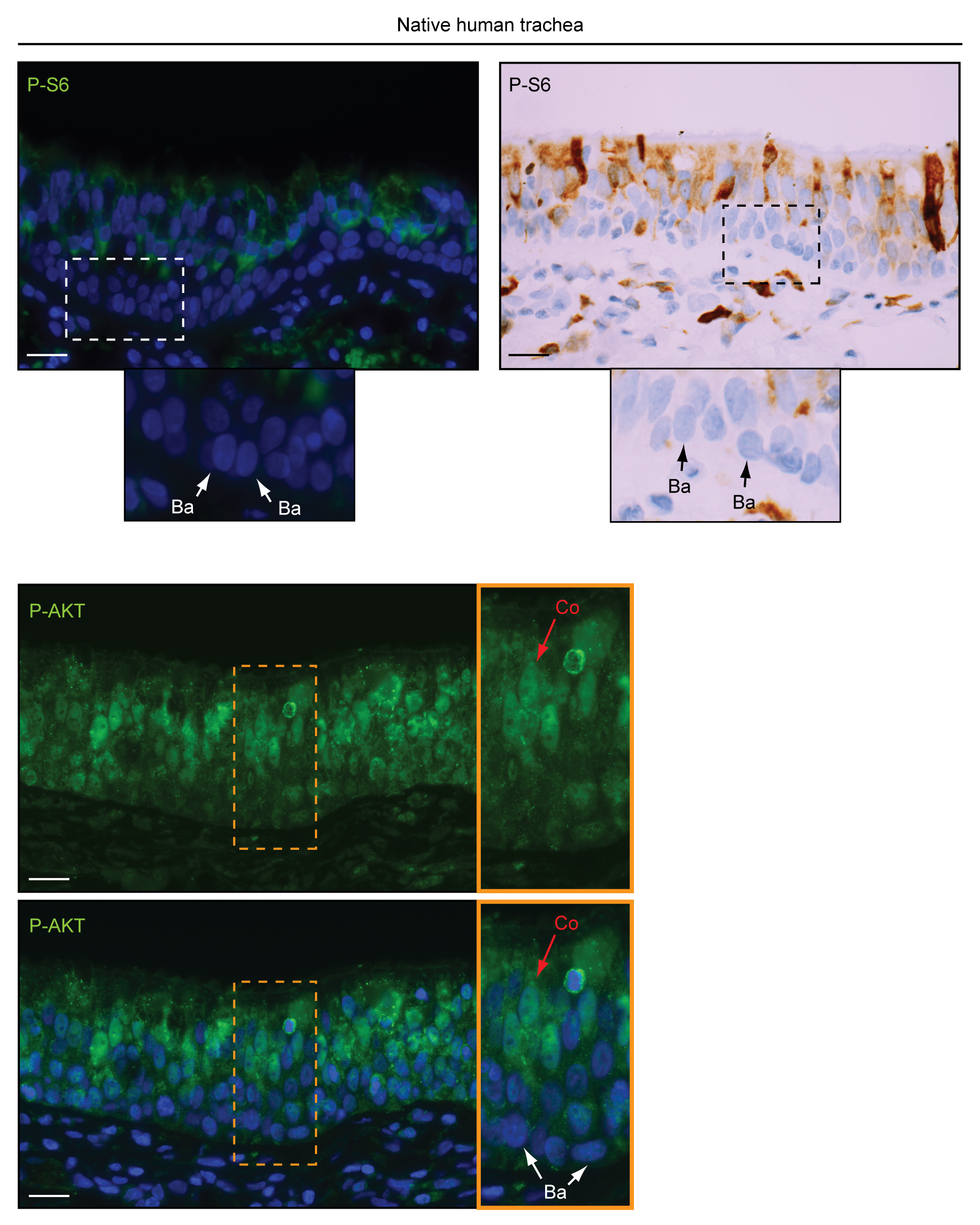

Supplement: S3 Fig — Tracheal tissue was stained for phospho-Ser240/244-S6 (P-S6) or phospho-Thr308-AKT (P-AKT). Insets correspond to magnified areas bounded by dashed boxes. White and black arrows point to representative basal cells (Ba). Representative columnar cells (Co) with nuclear P-AKT are indicated by red arrows. Scale bars are 20 μm. (TIF) [file pbio.1002581.s008.tif]

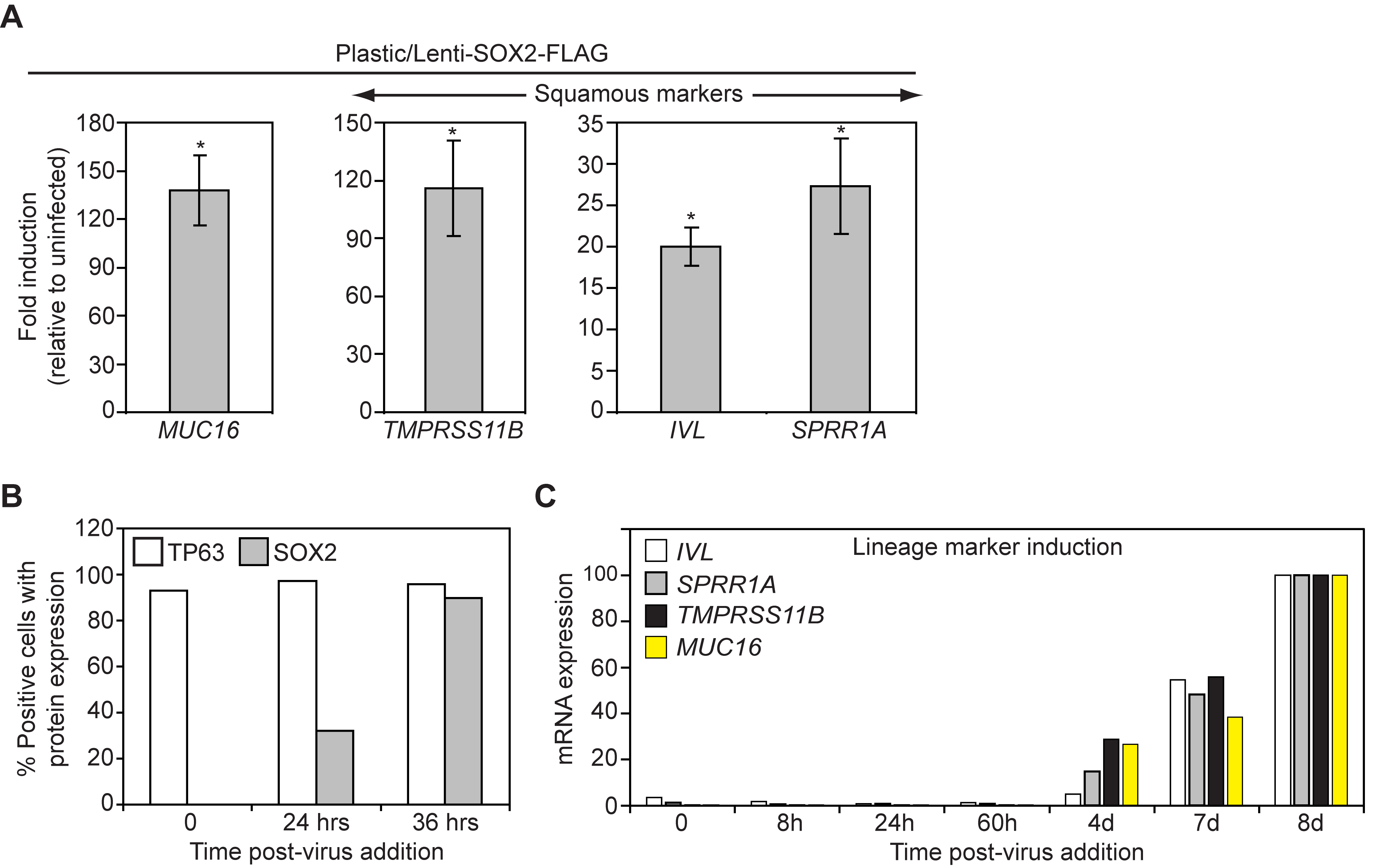

Supplement: S4 Fig — (A–C) Tracheobronchial basal cells growing on plastic were infected with control vector or Lenti-SOX2-FLAG and analyzed as indicated. (A) SOX2-FLAG induces markers of mucinous and squamous differentiation in basal cells. Five days following Lenti-SOX2-FLAG transduction, lineage marker expression was measured by qRT-PCR. Data are plotted relative to uninfected controls, which were assigned a value of 1 and generally had the same baseline marker expression as empty-vector infected cells. Means ± standard error of the mean (SEM) from three replicates are shown. Significance was calculated using paired two-tailed t tests. * p = 0.008 (MUC16), 0.04 (TMPRSS11B), 0.01 (IVL), 0.05 (SPRR1A). (B) Kinetic analysis of SOX2 and TP63 protein expression after Lenti-SOX2-FLAG transduction. Positive cells were identified by immunofluorescence staining, with 150–250 cells counted. (C) Time course of lineage marker induction following Lenti-SOX2-FLAG transduction. Marker expression was quantified by qRT-PCR. Data are plotted relative to the time point with the greatest amount of marker expression, which was assigned a value of 100. All plotted numerical data are in S2 Data. (TIF) [file pbio.1002581.s009.tif]

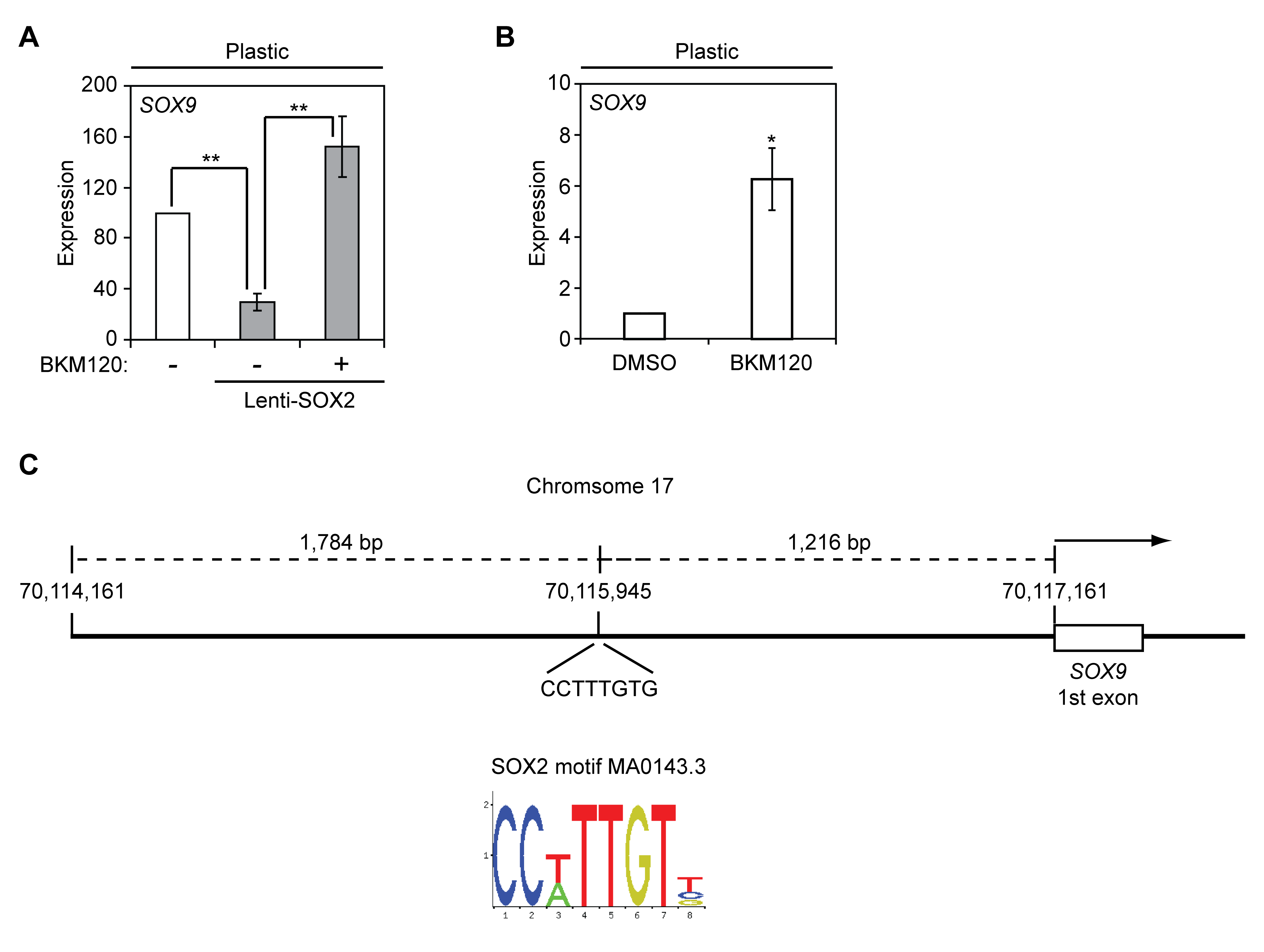

Supplement: S5 Fig — (A) Tracheobronchial basal cells growing on plastic were infected with control vector or Lenti-SOX2-FLAG ± 2.5 μM BKM120, and SOX9 levels quantified by qRT-PCR 36 hours post-infection. Data were normalized to expression in vector-transduced cells, which was assigned a value of 100. Means ± SEM from four replicates are shown. Significance was calculated using paired two-tailed t tests. **p = 0.003. (B) Tracheobronchial basal cells growing on plastic were treated with control DMSO vehicle or 2.5 μM BKM120. After 3 d, SOX9 expression was analyzed by qRT-PCR. Data were normalized to expression in DMSO-treated cultures, which was assigned a value of 1. Means ± SEM from three replicates are shown. Significance was calculated using a paired two-tailed t test. *p = 0.05. (C) Analysis of the human SOX9 promoter for SOX2 binding sites. 3,000 bp of genomic sequence upstream of the first SOX9 exon was scanned for the SOX2 motif MA0143.3 using the search tool at the JASPAR database (http://jaspar.genereg.net). A strong match was identified approximately 1,200 bp upstream of the first exon. All plotted numerical data are in S2 Data. (TIF) [file pbio.1002581.s010.tif]

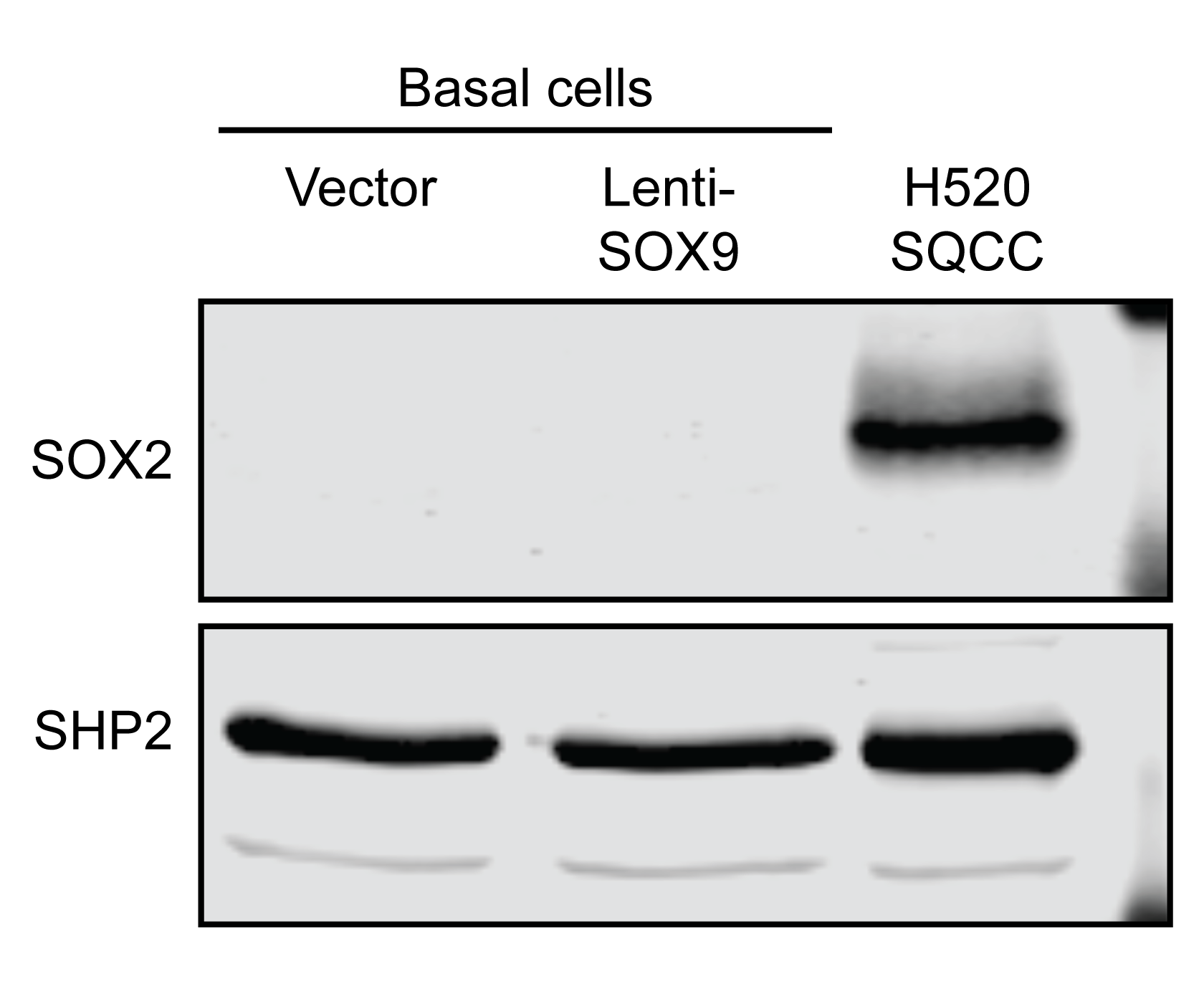

Supplement: S6 Fig — Basal cells proliferating on plastic were infected with control vector or Lenti-SOX9, and after 5 d, SOX2 expression was examined by immunoblotting 30 μg of lysate. The H520 SQCC cell line was used as a positive control. (TIF) [file pbio.1002581.s011.tif]

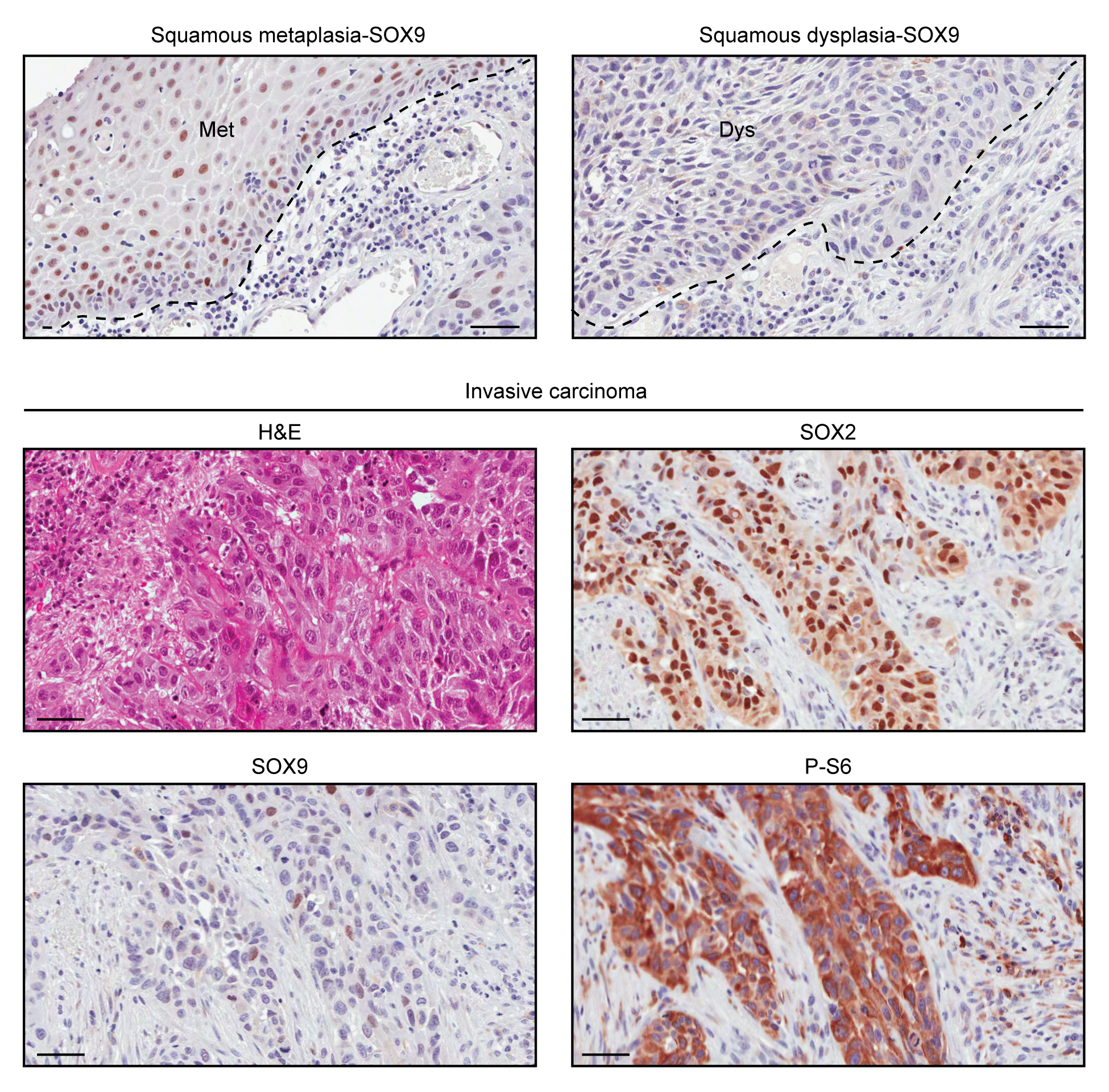

Supplement: S7 Fig — Larger areas of preneoplasia and representative areas of invasive disease from the lung resection shown in Fig 11. Sections were stained with the indicated antibodies. P-S6 = phospho-Ser240/244-S6. Dotted lines denote basolateral boundaries of metaplasia (Met) and dysplasia (Dys). Scale bars are 50 μm. (TIF) [file pbio.1002581.s012.tif]

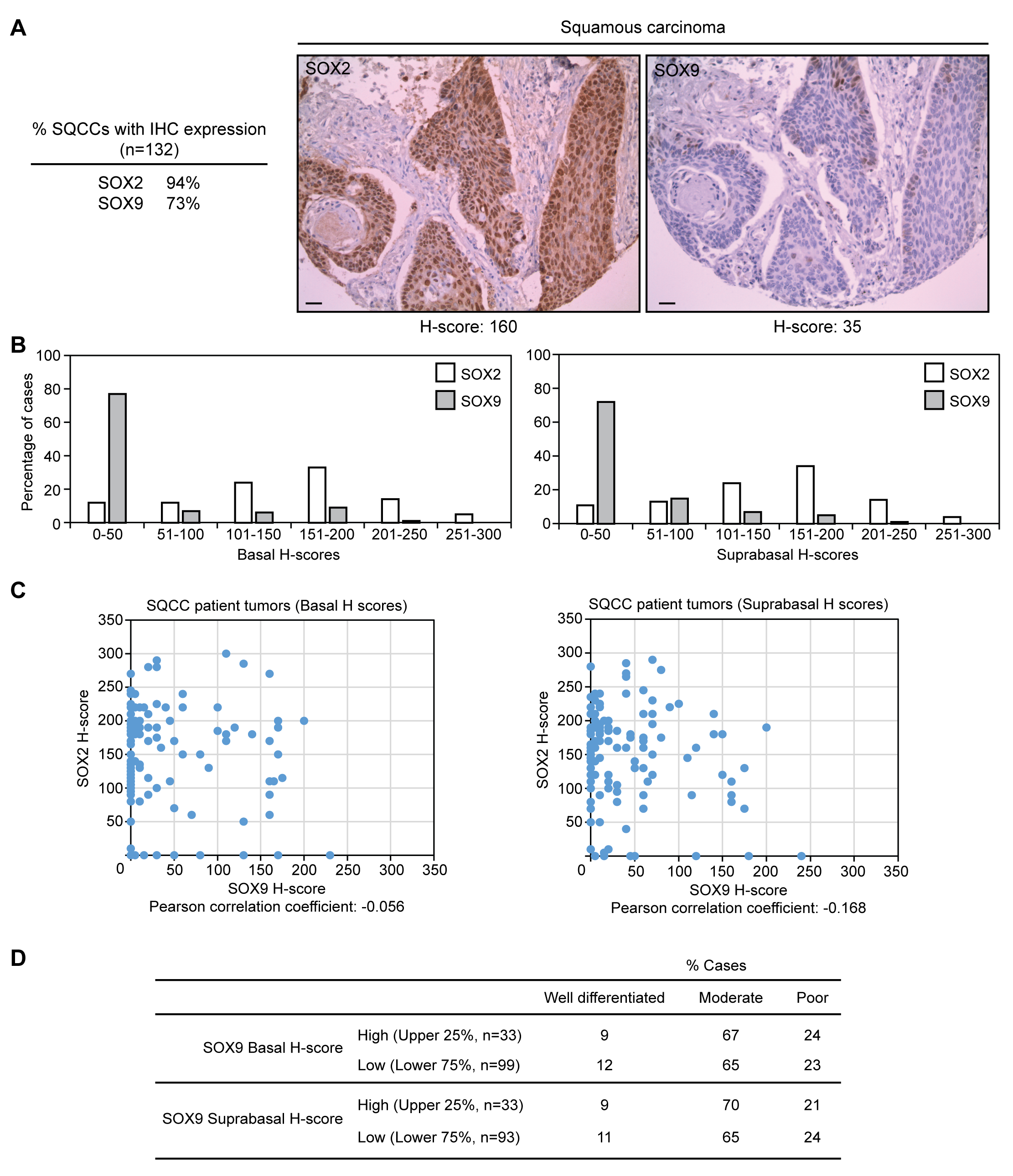

Supplement: S8 Fig — (A–C) IHC for SOX2 and SOX9 expression in a tissue microarray (TMA) derived from an SQCC cohort of 132 patients. (A) Representative SOX2 and SOX9 IHC in the TMA. Scale bars are 50 μm. (B) Distribution of SOX2 and SOX9 H-scores in the SQCC cohort. Data were derived from the TMA and each patient core was given an H-score for SOX2 and SOX9 expression (see also Table 1). The H-score was calculated for each core by summing: [(0 x % cells with no stain) + (1 x % cells with weak stain) + (2 x % cells with moderate staining) + (3 x % cells with strong staining)]. The H-score scale thus ranged from 0–300. Basal and suprabasal layers were scored separately with the hypothesis that in moderate and well-differentiated tumors, stem cells might reside in the basal layers and more differentiated progeny would be found in suprabasal areas. (C) Relationship between SOX2 and SOX9 protein expression in SQCC patients. (D) Comparison of number of cases by tumor grade in high versus low SOX9-expressors. All plotted numerical data are in S2 Data. (TIF) [file pbio.1002581.s013.tif]

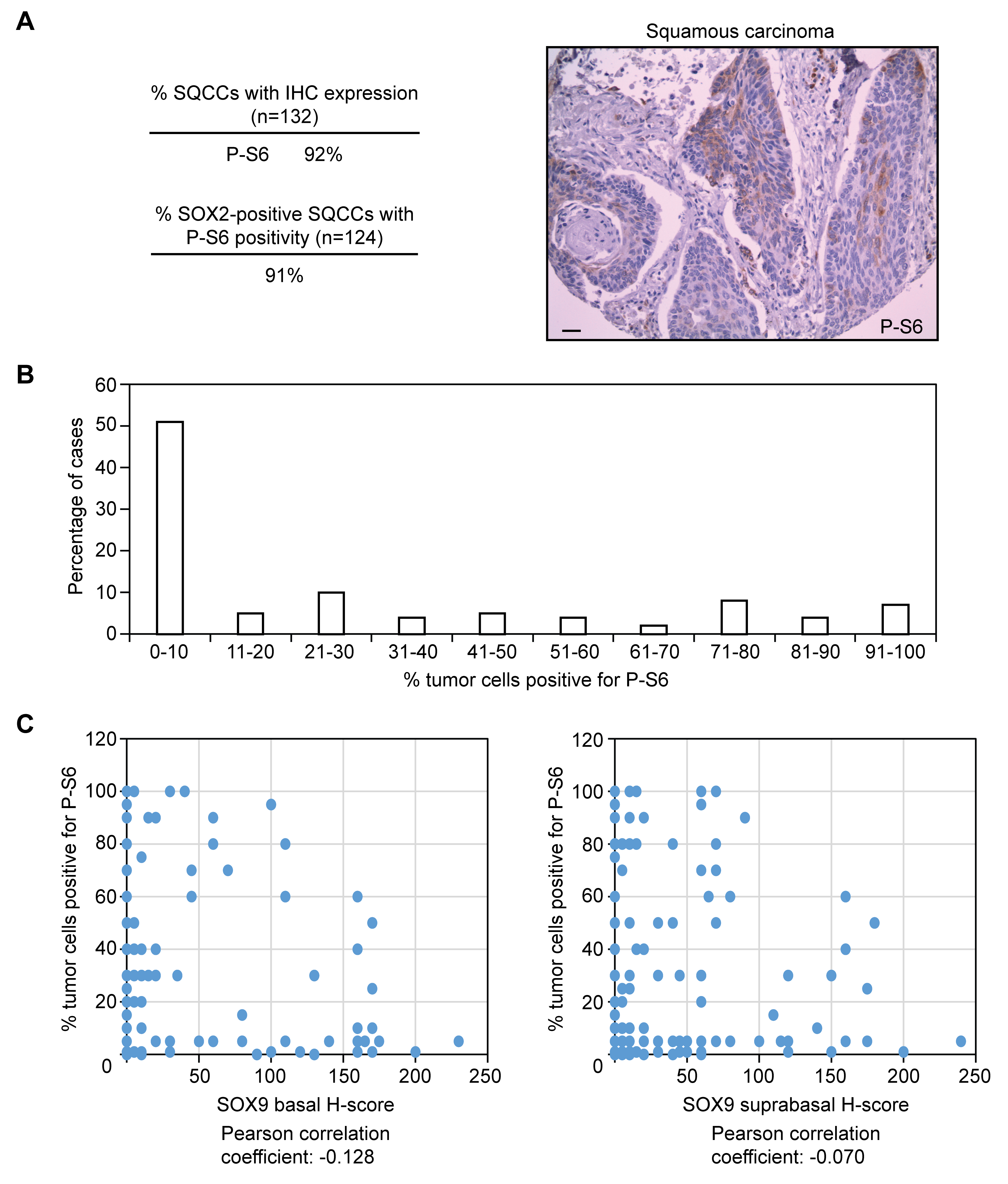

Supplement: S9 Fig — (A) Representative images of P-S6 IHC in a tissue microarray (TMA) derived from an SQCC cohort of 132 patients (same as S8 Fig). Scale bar is 50 μm. (B) Distribution of P-S6 expression data in the SQCC cohort. (C) Relationship between P-S6 and SOX9 expression in SQCC patients. All plotted numerical data are in S2 Data. (TIF) [file pbio.1002581.s014.tif]

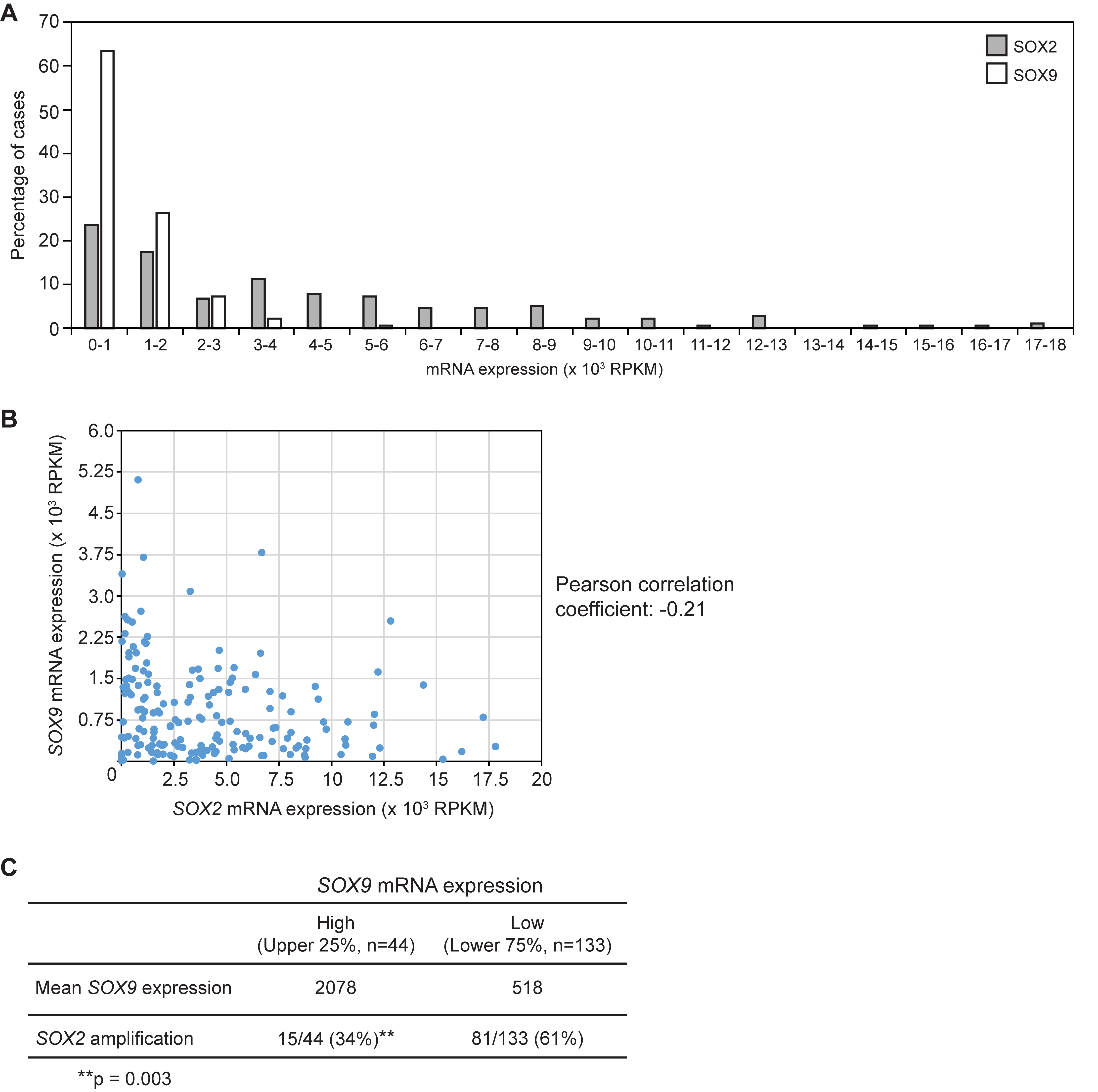

Supplement: S10 Fig — (A–C) All mRNA expression and copy number variation data are from the TCGA analysis of 177 primary SQCCs and are in S2 Data. (A) Distribution of mRNA expression across the patient cohort. RPKM = Reads Per Kilobase of transcript per Million mapped reads. (B) Relationship between SOX2 and SOX9 mRNA expression in SQCC patients. (C) Comparison of SOX9-high versus SOX9-low SQCCs and their associations with SOX2 amplification. Statistical significance was calculated using a two-tailed Fisher’s exact test. (TIF) [file pbio.1002581.s015.tif]
